# Transition-Metal-Doping of CaO as Catalyst for the OCM Reaction, a Reality Check

**DOI:** 10.3389/fchem.2022.768426

**Published:** 2022-02-11

**Authors:** Lukas Thum, Wiebke Riedel, Natasa Milojevic, Chengyue Guan, Annette Trunschke, Klaus-Peter Dinse, Thomas Risse, Reinhard Schomäcker, Robert Schlögl

**Affiliations:** ^1^ Technische Universität Berlin, Fakultät II, Institut für Chemie, Berlin, Germany; ^2^ Department of Inorganic Chemistry, Fritz-Haber-Institut der Max-Planck-Gesellschaft, Berlin, Germany; ^3^ Freie Universität Berlin, Institut für Chemie und Biochemie, Berlin, Germany; ^4^ BasCat—UniCat BASF JointLab, Technische Universität Berlin, Berlin, Germany; ^5^ Freie Universität Berlin, Institut für Experimentalphysik, Berlin, Germany

**Keywords:** oxidative coupling of methane, calcium oxide, doping, EPR, transition metal doping, heterogeneous catalysis

## Abstract

In this study, first-row transition metal-doped calcium oxide materials (Mn, Ni, Cr, Co., and Zn) were synthesized, characterized, and tested for the OCM reaction. Doped carbonate precursors were prepared by a co-precipitation method. The synthesis parameters were optimized to yield materials with a pure calcite phase, which was verified by XRD. EPR measurements on the doped CaO materials indicate a successful substitution of Ca^2+^ with transition metal ions in the CaO lattice. The materials were tested for their performance in the OCM reaction, where a beneficial effect towards selectivity and activity effect could be observed for Mn, Ni, and Zn-doped samples, where the selectivity of Co- and Cr-doped CaO was strongly reduced. The optimum doping concentration could be identified in the range of 0.04-0.10 atom%, showing the strongest decrease in the apparent activation energy, as well as the maximum increase in selectivity.

## Introduction

The selective activation of C-H bonds for the functionalization of hydrocarbons by oxidation reactions is a major challenge in catalysis. Although a large number of materials show high activity for these reactions, most of them also catalyze undesired side reactions that finally produce deep oxidation products, namely CO_2_ and water. A variety of metal oxides, either single or mixed oxides, represent the toolbox of the research field of oxidation catalysis. They are applied as catalysts as bulk materials or deposited as nanoparticles or monolayers at the surface of more or less inert support materials (Hermans et al., 2009; Sun et al., 2014; Schlögl, 2015). For an enhanced activity of the catalysts often a high specific surface is generated by high dispersion of the material. For a high selectivity, a very complex property profile is essential, which is governed mainly by the structural and electronic properties of the oxide material. In a first step, all oxides may be categorized as reducible or non-reducible under the reaction conditions. Reducible oxides provide lattice oxygen (O^2-^) from their surface or subsurface for oxygen insertion reactions or sites for the abstraction of a hydrogen atom in oxidative dehydrogenation reactions of hydrocarbons. This oxygen is considered as nucleophilic oxygen and can serve as selective species for the hydrocarbon oxidation reaction. Since the O^2-^ ion is extracted from the lattice to form a vacancy and the oxygen vacancies need to be replenished with gas-phase oxygen at a sufficiently fast rate to avoid the buildup of oxygen species which may cause undesired side reactions. This relation of the elementary steps of the reaction mechanism, that is assembled in the Mars-van-Krevelen mechanism, results in an overall reaction rate that is often independent of the oxygen partial pressure in the gaseous reaction mixture (Beck et al., 2012). Non-reducible oxides provide adsorption sites for oxygen molecules and control their activity by polarization. This occurs in particular at non-ideal surfaces, offering low coordinated sites e.g., steps and edges as adsorption sites (Schwach et al., 2015a; Schwach et al., 2015b). On the contrary, flat surfaces are almost inactive (Bajdich et al., 2015; Nilius and Freund, 2015; Aljama et al., 2018).

In this situation, different oxidation states of the oxygen molecule are possible, depending on the number and energy of transferred electrons. The resulting oxygen species are considered electrophilic and highly active for C-H activation. This difference in the selectivity behavior of reducible vs non-reducible oxides was nicely demonstrated by J. Haber and M. Witko (Haber and Witko, 2003). Due to the high activity, redox-active catalysts are also suitable for less reactive substrates like methane. For this reason, numerous reducible metal oxides have been tested as catalysts for the OCM reaction, one of the most promising but also most challenging reactions for a future methane utilization strategy (Zavyalova et al., 2011; Nguyen et al., 2020). Many non-reducible oxides also show activity for OCM, but with a rather low selectivity. The main reason for this observation is seen in the presence of different oxygen species with very different reactivity profiles. Therefore, many strategies have been tested for improving their performance in high throughput screening tests, rational approaches, and theoretical studies (Lee and Oyama, 1988; McFarland and Metiu, 2013; Zhang et al., 2020; Ohyama et al., 2021). The most promising approach for controlling the activation mechanism of oxygen and thereby the selectivity of OCM is the doping of the metal oxides with aliovalent ions, generating either a source or a sink for electrons within the material (Voskresenskaya et al., 1995).

Theoretically, wide-bandgap insulators are hardly able to activate oxygen (Bajdich et al., 2015). But in reality, it works due to the natural abundance of impurities (Voskresenskaya et al., 1995; Thum et al., 2019) causing strongly enhanced oxygen-binding on transition metals doped into CaO to form superoxo species. Here the O_2_^−^ binding energy depends on the oxidation state of the dopant, the lower it is, the stronger is the binding energy. Doping effects depend on the nature of the transition metals, e.g., Mo^2+^/Mo^3+^ has electron donor abilities in the near-surface region (top 3 ML) whereas Cr^3+^ in MgO is charge compensated by the formation of Mg^2+^ vacancies. Also, the cation vacancy formation energy can be drastically reduced by doping or the presence of low valance impurities (McFarland and Metiu, 2013). Mo is an especially good donor that can provide multiple electrons to surface adsorbates due to the high lying states of 4 d and 6s levels. The Cr^2+^→Cr^3+^ transition occurs spontaneously in MgO due to a lattice misfit serving as an intrinsic electron trap (Nilius and Freund, 2015).

Although there is experimental evidence for the beneficial impact of doping of alkaline earth metal oxides (AEMO) by alkaline metal oxides (AMO) or rare-earth metal oxides (REMO) on the oxidative coupling of methane (Lin et al., 1988; Kondratenko et al., 1999; Rane et al., 2006; Papa et al., 2010), the interpretation of such complex experimental results is challenging. For this reason, studies on model systems were done, investigating the properties of the dopants inside their matrices, the interaction of oxygen with the surfaces, and the electronic properties of the doped materials with theoretical and spectroscopic techniques. The electronic properties of oxide surfaces may be investigated by photoluminescence studies that allow monitoring of high energy defect sites (Stankic et al., 2006; Hao et al., 2013). The quenching of the luminescence signal by adsorbed oxygen is a sensitive probe for the interaction of the catalysts with oxygen (Bengoechea-Encabo et al., 2016; Puust et al., 2017). Deeper insight into the doping effects is obtained from theoretical studies that allow a prediction of the key descriptors of the oxidation reactions (Hu et al., 2011; Sun et al., 2013; Bajdich et al., 2015). The study by the Norskov group demonstrates the influence of doping of AEMOs with traces of transition metal ions (TM) on the binding energy of hydrogen atoms and methyl groups as such descriptors. The derived volcano plot shows a strong impact and room for improvement for the activity of the investigated catalysts by TM doping. Although this study was only focused on the activity it also suggests changes in the selectivity caused by doping (Aljama et al., 2018). Another contribution to the understanding of doping effects was published by Freund and co-workers. They showed by STM studies how doping facilitates the dissociative adsorption of oxygen already at ambient temperatures (Nilius and Freund, 2015). To demonstrate the relevance of the model studies for real catalysts, a series of TM doped CaO samples was prepared with different dopant metals and concentrations. Due to the choice of CaO for the model studies, it was also chosen here, although pristine CaO is known to be a poor OCM catalyst. Hence, it should be particularly easy to identify positive effects of doping on OCM performance.

Photoluminescence spectroscopy was applied to gain insight into the effect of doping on electronic properties. EPR measurements allow for the investigation of the environment of the dopant ions within the CaO matrix. The catalytic studies were focused on the selectivity, especially the ratio of selective oxidation to deep oxidation. In order to link these experiments to the model studies the essential research questions of this study were:

How are the dopants dispersed within the CaO matrix?

Where are the dopants located?

Do the dopants change the surface or bulk properties of CaO?

Do dopants contribute to additional redox-active sites at the surface?

What is the impact of doping on catalyst selectivity?

## Experimental

Synthesis. Transition metal-doped calcium carbonates (M_x_Ca_1-x_CO_3_, with 0.05–3.2%) were prepared by co-precipitation as oxide precursors in a similar manner as described before for pure CaO.^14^ A mixed metal nitrate solution containing 10.7 wt% calcium nitrate (Ca(NO_3_)_2_ × 4H_2_O, Fisherscientific analytical reagent grade) and the appropriate amount of transition metal nitrate (Cr^3+^, Mn^2+^, Ni^2+^, Co^3+^, Zn^2+^, Acros Organics) and an 8.03 wt% ammonium carbonate solution ((NH_4_)_2_CO_3_, Roth, p. a.) were prepared and simultaneously added over a period of 1 hour to a 0.74 wt% ammonium carbonate solution at 25°C under constant stirring using an automated LabMax® reactor (Mettler Toledo). The product was aged in the solution for 1 h up to 72 h at 25°C, filtered, washed multiple times with millipore water, and dried at 65°C for 24 h in static air. The calcination was performed, if not mentioned otherwise, in the corresponding setup, before the experiments. For catalytic measurements the resulting powder was pressed at 200 bar, crushed, and sieved to yield a sieve fraction of 200–300 µm. As a reference sample (ref. CaO), the synthesis was performed with no additional metal nitrate solution and a commercial sample was prepared (pure CaO, Alfa Aesar Puratronic 99.998%).

XRD Analysis. XRD analysis was performed using a STOE STADI P transmission diffractometer equipped with a Cu anode, primary Ge (111) monochromator, and a Dectris MYTHEN 1K position-sensitive microstrip solid-state detector. The samples were crushed before analysis using an agate mortar. The data were fitted using the Bruker TOPAS software.

Nitrogen adsorption. An Autosorb^®^ iQ by Quantachrome^®^ Instruments was used to determine the surface area of the precipitated carbonates, using a 7-Point BET method assuming 0.162 nm^2^ as the nitrogen cross-sectional area. The samples were degassed at 200°C prior to the measurements.

Elemental Analysis. The transition metal contents and impurities were determined by ICP-OES measurements using a Varian ICP-OES 715 ES. Depending on the metal content 50 mg up to 500 mg mixed carbonates were dissolved with 3 ml 69% HNO_3_ (ROTIPURAN® Supra, ROTH) and diluted with water (VWR, ultrapure, HPLC grade) to a total volume of 15 ml. A dilution series of an ICP standard (Periodic table mix one for ICP, TraceCERT®) was used for calibration. The main impurity was found to be Mg followed by Sr with 150 ppm for all samples. Sodium impurities were found to be below the detection limit (detection limit 50 ppm).

Thermogravimetric Analysis. Using an STA 449 Jupiter thermoanalyser (Netsch) equipped with the pulse box for PulseTA, 10 mg of the carbonate samples were decomposed under a constant flow of synthetic air (20.5% O_2_ in N_2_) applying a heating rate of 2°C/min up to 1,000°C.

Catalytic Tests. For the catalytic test 50 mg of carbonate, the precursor was diluted in 750 mg of silicon carbide and fixed with quartz wool in a 4 mm inner diameter quartz tube reactor resulting in a bed length of 40 mm. The catalysts were tested in automated reactor units testing six to eight catalysts in parallel. The analysis of the reaction products was achieved using a GC equipped with TCD and FID detectors. Prior to the catalytic testing, the catalysts were activated in the reactor at 800 °C in synthetic air (N_2_: O_2_, 4:1, 40 ml/min) for 6 h. Applying a fixed feed composition (3:3:1 N_2_:CH_4_: O_2_, if not indicated otherwise), the total flow was varied between 37.5 ml/min up to 200 ml/min and the temperature between 650 and 800°C. Methane conversion (*X*_*CH4*_) and selectivities (*S*_*i*_) were calculated using Eq. 1 and Eq. 2, respectively, with *x*_*i*_ the molar fraction of compound i, *ν* the stoichiometric factor, and *p* the index for the reaction products (C_2_H_6_, C_2_H_4_, CO, and CO_2_).

$$X_{CH_{4}}=1-\frac{x_{CH_{4}}}{x_{CH_{4}}+\sum \frac{1}{\nu_{p}}x_{p}}$$ (1)

$$S_{i}=\frac{\frac{1}{\nu_{i}}x_{i}}{\sum \frac{1}{\nu_{p}}x_{p}}$$ (2)

Photoluminescence. Photoluminescence spectra were recorded using a Varian LS-55 Fluorescence Spectrometer. Calcium carbonate samples were placed in a quartz cuvette attached to a vacuum and gas supply line. For preparation the samples were heated under dynamic vacuum to 900°C for 6 h applying a rate of 5°C/min, reaching a vacuum of less than 5 × 10^−6^ mbar. For quenching experiments, oxygen was introduced to the sample. For some experiments, the samples could be sealed in the cuvette by melting the quartz tube feed using a torch.

EPR Spectroscopy. The oxide samples for EPR measurements were all prepared similarly to the PL samples avoiding contact with air. 2.9 mm EPR tubes made of high purity quartz were attached to a quartz flange and connected to a vacuum line. The catalyst was loaded into the tubes up to a bed height of 0.5–1.0 cm, degassed, and heated under dynamic vacuum (<5 × 10^−6^ mbar) to 900°C for 6 h, applying a heating rate of 5°C/min. After cooling down, the samples were sealed under vacuum with a torch. In addition, the carbonate samples were measured as prepared without any activation process.

Two samples comparing the pre- and post-reaction condition of the catalyst were prepared in a reactor setup suitable for the OCM reaction, equipped with four MFCs (Bronkhorst), providing methane, nitrogen, oxygen, and helium, a GC, and a folding oven. Reactors were prepared from a 2.9 mm outer diameter EPR tube with 6 mm outer diameter quartz tubes attached to fit in the catalytical setup. The carbonate sample was diluted with 0.4 mm quartz spheres and fixed in the reactor with quartz wool. The silicon carbide diluent used in the catalytic tests was found to disturb the EPR measurements. The amount of catalyst was chosen to yield a bed length of roughly 1 cm. Both samples were activated at 900°C for 6 h in synthetic air (20% O_2_ in N_2_) and cooled to 750°C. For the pre-reaction sample, the feed was switched to helium, cooled down to room temperature, and both ends of the 2.9 mm tube were sealed with a torch. For the post-reaction sample, the feed was switched to an OCM feed of 4:4:1 (CH_4_:N_2_:O_2_) and kept on stream for 12 h. The feed was again switched to helium, the sample was cooled down and sealed, to avoid contact to air.

Continuous-wave (cw) EPR measurements at room temperature were conducted at 9.8 and 34 GHz microwave (mw) frequencies employing a Bruker B-ER420 spectrometer upgraded with a Bruker ECS 041XG microwave bridge and a lock-in amplifier (Bruker ER023M) using a Bruker SHQ resonator for 9.8 GHz and an ER051QT resonator for 34 GHz measurements. Echo-detected pulsed EPR measurements at 34 GHz were conducted on a Bruker ElexSys 580 setup with a home-built cavity (F. Lendtzian, TU Berlin). Field swept echo (FSE) spectra at 34 GHz were obtained at 20 K applying a two-pulse mw sequence (16-250-32 ns). Transient nutation (TN) measurements at 34 GHz were conducted applying a PEANUT (Phase-inverted Echo-Amplitude detected Nutation) pulse mw sequence with a π/2 pulse length of 32 ns, a delay time τ of 250 ns, and a high turning angle (HTA_x_) pulse of 2048 ns incrementing the phase inversion time within the HTA_x_ pulse by 2 ns starting with an initial inversion after 32 ns (Stoll et al., 1998).

## Results

### Synthesis and Characterization

For the synthesis of doped calcium oxide materials, carbonates were chosen as suitable precursors due to their better resistance towards moisture and carbon dioxide in ambient atmosphere compared to calcium oxides which rapidly react to form hydroxides and carbonates. Nitrate precursors were used as metal sources and ammonium carbonate as the precipitating agent to avoid alkaline impurities in the materials, which can highly affect the catalytic performance (Kondratenko et al., 1999; Rane et al., 2008; Arndt et al., 2011; Elkins et al., 2016). The precipitation temperature was precisely regulated to 25°C with the automated precipitation reactor to exclude the formation of an aragonite phase during the synthesis, which is favored to be formed at 29°C (Holleman et al., 2007).

To get a first impression regarding the catalytic effect of different dopants, Cr^3+^, Mn^2+^, Co^3+^, Ni^2+,^ and Zn^2+^ nitrates were co-precipitated to yield M_x_Ca_(1-x)_CO_3_ with x being 0.1% applying an aging time of 1 h, the results can be found in Table 1. The synthesis was then optimized for the Mn and Ni-doped samples in terms of dopant concentration and phase purity of the carbonate precursors.

**TABLE 1:** Results of OCM tests using transition metal-doped CaO catalysts (50 mg cat, 750 mg SiC, 50 ml/min, CH_4_:N_2_:O_2_ = 3:3:1).

				700°C			750°C			800°C		
Catalyst	ID*	x_i_ (mol%)**	E_A_^***^ (kJ/mol)	X (CH_4_) (%)	S(C_2+_) (%)	S(CO_x_) (%)	X (CH_4_) (%)	S(C_2+_) (%)	S(CO_x_) (%)	X (CH_4_) (%)	S(C_2+_) (%)	S(CO_x_) (%)
CaO	24,254	—	131	3.7	3.8	96.2	8.6	9.0	91.0	17.4	16.5	83.1
CaO:Cr	24,414	0.067	108	6.6	0.4	99.6	14.0	0.5	99.5	18.0	0.7	99.3
CaO:Mn	24,256	0.029	102	7.9	5.0	95.0	14.4	12.4	87.4	21.7	19.7	79.7
CaO:Co.	24,412	0.074	123	7.6	2.8	97.2	14.3	6.1	93.9	19.7	9.6	90.2
CaO:Ni	24,413	0.062	117	5.7	5.5	94.5	12.1	13.6	86.2	21.5	24.4	74.7
CaO:Zn	25,054	0.063	127	4.1	5.8	94.2	8.8	11.4	88.6	16.7	20.2	79.3

*internal sample ID, **determined by ICP-OES, ***calculated from data shown in Supplementary Figure S14.

**FIGURE 1:**
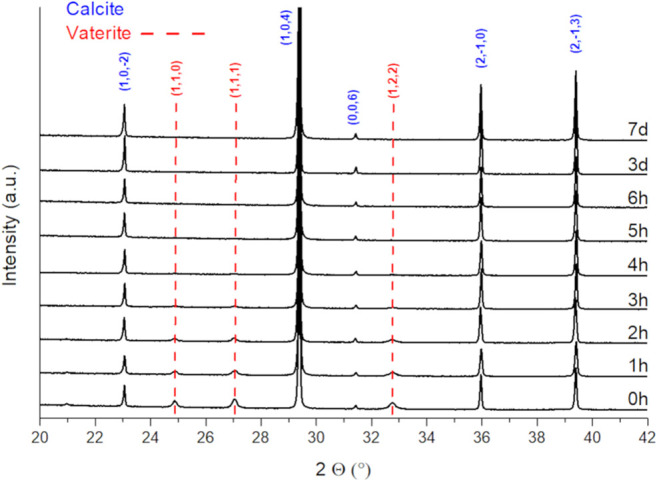
XRD patterns of precipitated and dried CaCO_3_ prepared applying different aging times.

To obtain phase pure precursor materials, the aging time after precipitation has been varied from 1 h up to 7 days. XRD analysis of the precipitates (Figure 1) reveals two carbonate allotropes being formed during synthesis: vaterite and calcite. Aragonite, being thermodynamically favored at elevated pressures or temperatures, was not found. The samples exhibit a high crystallinity, and the presence of amorphous phases is excluded. The drying temperature of 65°C was applied to prevent the formation of an aragonite phase during the drying process. Upon aging, the metastable vaterite is progressively converted into the thermodynamically favored calcite structure (Nebel and Epple, 2008). After 4 h no phase next to calcite could be observed. The initial formation of vaterite can be explained by the presence of ammonia in the solution, which is known to promote the formation of vaterite instead of calcite (Holleman et al., 2007). To ensure a complete phase transition towards thermodynamically stable calcite, an aging time of 24 h was chosen for the doped samples.

In Table 2, the concentrations determined by ICP-OES and the surface areas of the precipitates are presented. All precipitates exhibit a very small surface area below 1 m^2^/g which varies with no clear trend. It is also noteworthy, that a lot of doping metal is lost during synthesis (remains in the mother liquor) and is not incorporated into the host lattice. In the case of manganese doped calcium carbonate, roughly half of the transition metal is lost during synthesis, wherein in the case of nickel the loss increases with increasing nominal Ni content. Since nickel is known to form stable amine complexes (van Geet, 1968), the loss is likely a result of the precipitating agent chosen. The high concentration of ammonia and the low concentration of transition metals in solution results in incomplete precipitation of the transition metal.

**TABLE 2:** Synthesis data of precipitated carbonates.

Dopant	Ni			Mn				
*x*_*i*_ target (mol%)	*x*_i_* (mol%)	*S*_*BET*_ (m^2^/g)	ID***	*x*_*i*_ * (mol%)	*S*_*BET*_ (m^2^/g)	a** (Å)	c** (Å)	ID***
ref	0	1.7	24,254	0	1.7	—	—	24,254
0.05	0.02	0.42	26,443	0.02	1.2	4.992	17.071	25,815
0.10	0.04	0.47	26,444	0.04	1.1	4.991	17.067	25,816
0.20	0.08	0.22	26,445	0.08	—	4.990	17.045	26,068
0.40	0.16	0.28	26,446	0.17	0.79	4.991	17.067	26,070
0.80	0.29	0.62	26,447	0.36	0.79	4.989	17.060	26,071
1.60	0.38	—	26,448	0.69	1.3	4.987	17.005	26,072
3.20	—	—	—	1.41	—	4.990	17.067	26,073

*determined by ICP- OES, **determined by the fit of XRD, data,***internal sample ID.

**FIGURE 2:**
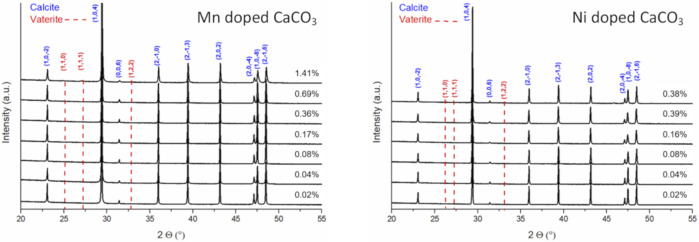
XRD patterns of precipitated and dried Mn (left) and Ni (right) doped CaCO_3_ aged for 1 day.

Inspecting the XRD patterns of the resulting materials no additional signals indicating segregation of the dopants during carbonate synthesis were observed (Figure 2). Fitting the XRD data of the manganese series, a slight lattice contraction can be observed hinting at an insertion of transition metals into the calcite lattice, though the total change of the lattice constants (*a* and *c*) is less than 0.1% (Table 2). In the case of the highest nickel doped carbonate, hints of vaterite can be found even after aging for 24 h (Supplementary Figure S2).

To investigate the influence of the doping on the carbonate decomposition, exemplary manganese doped samples were decomposed under synthetic air using thermogravimetric analysis (Supplementary Figure S1). The theoretical mass loss was determined, assuming a molar mass of 100.1 g/mol for the carbonates and 56.1 g/mol for calcium oxide. For all three samples, the decomposition starts around 550°C and is completed at 730°C, which is in good agreement with the literature (start 540°C) (Holleman et al., 2007). No major impact of doping on carbonate decomposition can be observed. To inspect a phase separation of the doping metal during decomposition of the carbonate, the highest doped sample (1.41 atom% (Mn,Ca)CO_3_) was decomposed in a transfer holder allowing a glove box handling of the material. XRD analysis did not reveal additional signals, but a lattice contraction could be determined implying an incorporation of the doping metal into the calcium oxide lattice (Supplementary Figure S3).

To analyze the impact of the dopant on the electronic structure of the catalyst surface, photoluminescence spectroscopy was conducted, which has been proven to be a useful tool in the surface characterization of alkaline Earth oxides (Coluccia et al., 1978; MacLean and Duley, 1984; Stankic et al., 2006; Schwach et al., 2015a). Due to the large bandgap of CaO, irradiation with UV light (>200 nm) can exclusively excite under-coordinated surface ions (Abrams and Holloway, 2004; Stankic et al., 2006). To exclude influences from adsorbates or hydroxyl groups, all samples were measured under vacuum (*p* < 5 × 10^−6^ mbar) and were annealed in a vacuum at 900°C for 6 h before the experiments.

**FIGURE 3:**
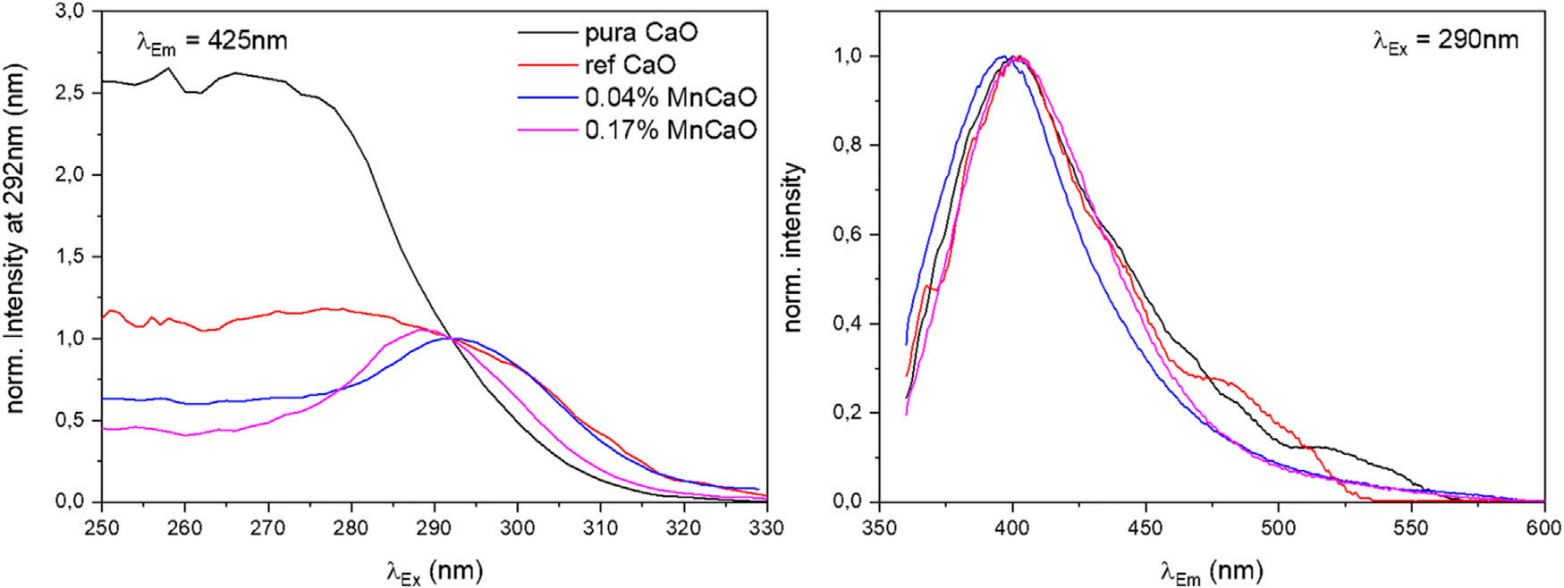
Normalized excitation and emission spectra at λ_Em_ = 425 nm and λ_Ex_ = 290 nm of pure CaO and Mn-doped CaO samples under vacuum.

It has to be mentioned, that the overall photoluminescence intensity differed strongly for different samples (compare Supplementary Figure S7). In turn, the spectrometer settings had to be adjusted which renders a quantitative comparison of the recorded spectra difficult. In general, it was observed that pure samples not containing any dopants exhibited broad, intense signals compared to doped samples (Figure 3, pure sample). Adding a high concentration (>0.17 atom%) of transition metals to CaO resulted in a complete loss of the signals (Supplementary Figure S8 and Supplementary Figure S9). It also has to be mentioned that the sample containing Zn was not stable and metallic Zn evaporated during pretreatment at the high temperatures in a vacuum.

Comparison of the excitation spectra of pure and Mn-doped CaO (Figure 3, Supplementary Figure S10), shows a significant change in the shape of excitation spectra, whereas the emission spectra appear to be almost unaffected. In general, a maximum of emission can be found around λ_Em_ = 400 nm for all samples which is hardly affected by the applied excitation energy and in accordance with results reported for CaO nanocrystals (Stankic et al., 2006). The excitation spectra, however, are strongly affected by the doping where the undoped samples (pure and ref CaO) exhibit broad intense spectra with no clear maximum, while the doped samples exhibit a clear maximum around λ_Ex_ = 290 nm (compare Figure S9) which can be attributed to surface edge sites (lit. O_4C_^2-^ 4.4 eV, λ_Ex_ = 280 nm) (Garrone et al., 1980). The deviation from the literature values might be explained by particle size dependence of the excitation energy (Stankic et al., 2005). The broader spectrum of the pure CaO sample (pure CaO) can be explained by the contribution of luminescence from low index planes (lit. O_5C_^2-^ 5.3–5.5 eV, λ_Ex_ = 225–235 nm) (Garrone et al., 1980; Stankic et al., 2006), as well as by better spatial isolation of the luminescent sites in the doped oxides due to their reduced abundance. Luminescence derived from corner sites was not observed in any sample (lit. O_3C_^2-^ 3.75 eV, λ_Ex_ = 330 nm) (Stankic et al., 2006).

**FIGURE 4:**
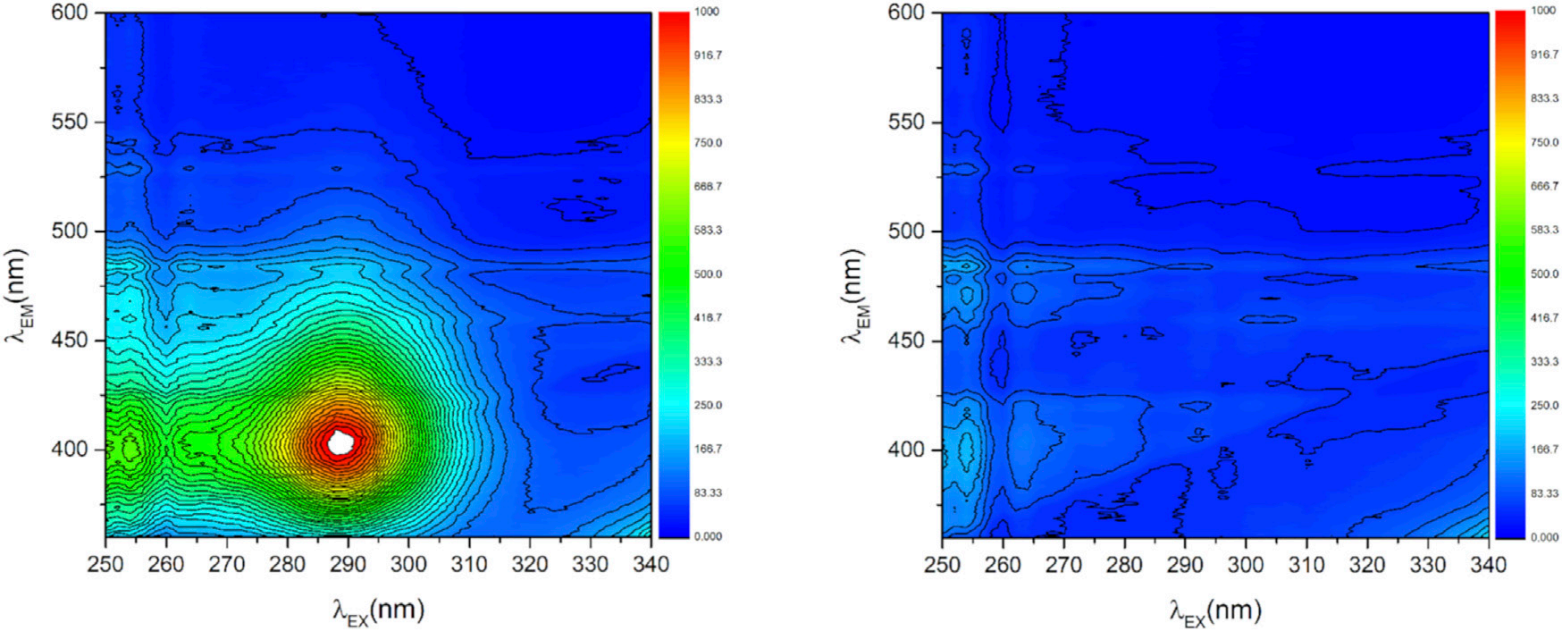
2D photoluminescence spectra of Mn doped CaO (0.17 atom% Mn) in vacuum (*p* < 5*10^−6^ mbar, left) and exposed to 1 mbar O_2_ (right).

Upon oxygen exposure, the photoluminescence could be completely quenched (see Supplementary Figure S7, fourth sample (ref CaO), 10 mbar O_2_). Figure 4 shows as an example the full 2D photoluminescence spectra recorded at excitation wavelengths between 250 and 340 nm and emission wavelengths between 360 and 600 nm of the 0.17 atom% Mn-doped CaO sample recorded before and after addition of oxygen to the sample. In vacuum one prominent signal can be observed at λ_Em_ = 400 nm and λ_Ex_ = 290 nm (Figure 4, left). Upon adding 1 mbar oxygen to the sample the signal is completely quenched (Figure 4, right), proving that the recorded photoluminescence is still being exclusively related to surface sites and no bulk luminescent site was created by the dopant (Bengoechea-Encabo et al., 2016).

**FIGURE 5:**
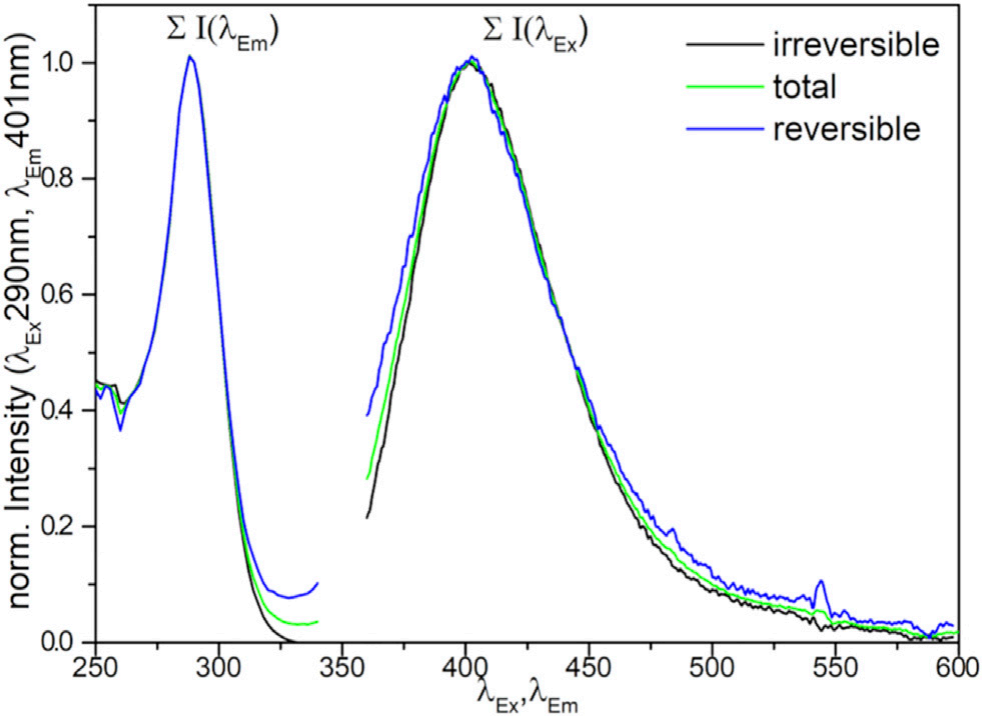
Normalized loss of photoluminescence (difference spectra of 0.17 atom% Mn doped CaO on exposure to oxygen (total) and after reapplying vacuum (reversible).

Reapplying vacuum resulted in regeneration of the signal, proving the reversibility of the process, though not the complete signal was retrieved (see Figure S11). Comparing the signals before the addition of O_2_ and after reapplying vacuum (Figure 5) shows no difference in the shape of the obtained spectra, indicating no difference in the nature of the reversible and irreversible quenched sites, proving reversibility of the catalyst-oxygen interaction even on the doped samples.

The Raman spectra show no signals (Supplementary Figure S12). While the lack of Raman signals is expected for fcc lattices, the spectra provide no evidence for adsorbed oxygen species such as O_2_^2-^ or molecular adsorbed oxygen (Xiao-Dong et al., 1991). This supports the idea of quenching caused by the collision of oxygen molecules with the catalyst surface. The absence of a Raman signal is also proof of the clean surface of the material. No indications for residual carboxyl groups from the synthesis can be found.

**FIGURE 6:**
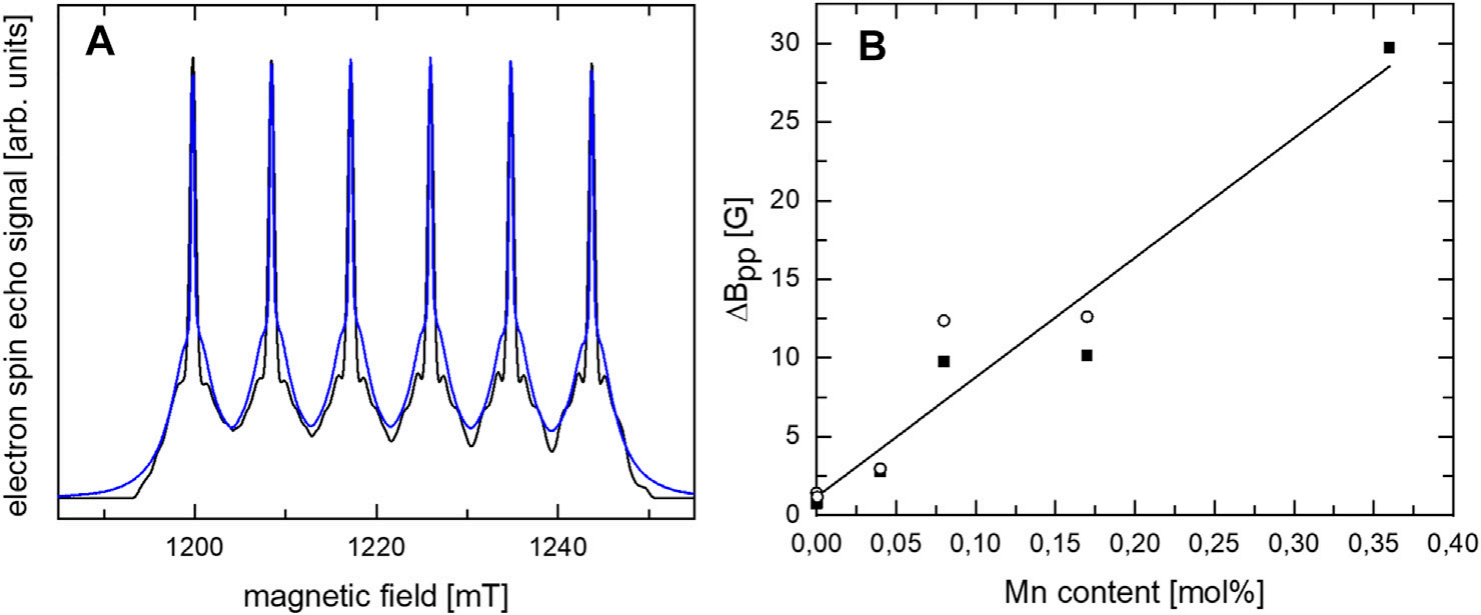
**(A)** 34 GHz FSE spectra of CaO with 0.04 mol% Mn (blue) and simulation (black) assuming a cubic symmetry. **(B)** Dependence of ΔB_pp_ linewidth in 34 GHz (open circles) and 9.8 GHz (solid squares) cw EPR measurements at room temperature on the Mn content.

For a further understanding of the Mn-doped CaO samples, EPR measurements were conducted. The 34 GHz field swept echo (FSE) measurements of the CaO sample with 0.04 mol% Mn shown in Figure 6A display intense EPR signals which not only exhibit the narrow six-line pattern (I_Mn_ = 5/2) originating from transitions between the m_s_ = ±1/2 sublevels, but also the “pedestal” powder pattern arising from the remaining orientation-dependent transitions in the S = 5/2 spin state of Mn^2+^ as verified by transient nutation EPR measurements (see Figure S4). The narrow width (10 mT) of this powder pattern proves that Mn^2+^ is substituting for Ca at this site of octahedral symmetry. Measuring at 34 GHz, no change in width of the narrow transitions is observed, in agreement with the expectation of negligible g matrix anisotropy of Mn^2+^ at high symmetry sites. The spectrum can be simulated assuming the restricted spin Hamiltonian parameter set expected for octahedral site symmetry, i.e., all second rank tensor elements vanishing, leading to isotropic hyperfine interaction (hfi) and g matrix, and only two elements of the fourth rank zero field splitting tensor are non-vanishing, related by the condition B_44_ = 5B_40_. In accordance with this prediction the spectrum can be fitted by: A = 242.3 MHz, g = 2.000, B_44_ = 2.8 MHz, B_40_ = 0.56 MHz B_40_ compares well with the value 0.482 MHz determined for cubic Mn:ZnSe by Cavenett (Cavenett, 1964).

For increasing Mn content, the ΔB_pp_ linewidth in 9.8 and 34 GHz continuous wave (cw) EPR measurements at room temperature was found to increase nearly linearly with increasing Mn content (see Figure 6B and Supplementary Figure S6). A linear increase in ΔB_pp_ linewidth due to dipol-dipol interactions is expected for decreasing average distances of the Mn ions in case of a homogeneous distribution. No indication for a significant deviation from the linear dependence caused by exchange interaction between “clustered” ions is detected. The nearly linear increase in ΔB_pp_ linewidth with increasing Mn content attests to a rather homogeneous Mn distribution in the CaO and no clear clustering in the investigated range up to 0.36 mol% Mn which is in agreement with results by de Biasi and Grillo for Mn-doped CaO (Biasi and Grillo, 1999). 9.8 GHz cw EPR measurements of the 0.04 atom% doped CaO at room temperature before and after catalysis (Supplementary Figure S5) showed no indication for a change of the site symmetry of Mn.

### Catalytic Experiments

In a first screening, different first-row transition metals were tested as potential dopants for calcium oxide. The OCM testing conditions were chosen to avoid full conversion of oxygen over a wide parameter range of temperatures and flow rates. Due to the comparatively oxygen-rich feed (3:3:1, CH_4_:N_2_:O_2_), the overall C_2_ selectivities are rather low. If the mechanism of Lunsford et al. is assumed, methyl radicals are released from the catalyst surface and upon a combination of two radicals, ethane is formed (Ito et al., 1985). Therefore, a high oxygen concentration in the gas phase promotes the collision of the methyl radicals with the oxygen di-radical resulting in deep oxidation products. On the contrary, full oxygen conversion would result in a loss of information and strongly decreases the comparability of the results. Before varying temperature and total gas flow, the catalyst was held on stream for 150 h to reach a stable catalytic performance (Supplementary Figure S13). Alkaline earth oxides are known to sinter at high temperatures altering the surface area which results in decreasing activity over time (Müller et al., 2008; Zhu et al., 2011). This is enhanced by the combustion products water and carbon dioxide (Borgwardt, 1989). In Table 1 the results of the catalytic testing with different doping metals are summarized. The doping of calcium oxide has a significant influence on the catalytic performance, depending on the used transition metal. Except for Zn, all other transition metals decrease the apparent activation energy significantly and enhance the reaction rates. The largest effect for the decrease of the activation energy is observed for manganese as dopant, reducing the activation energy from 131 kJ/mol to 102 kJ/mol, followed by Cr, Ni, and Co.

**FIGURE 7:**
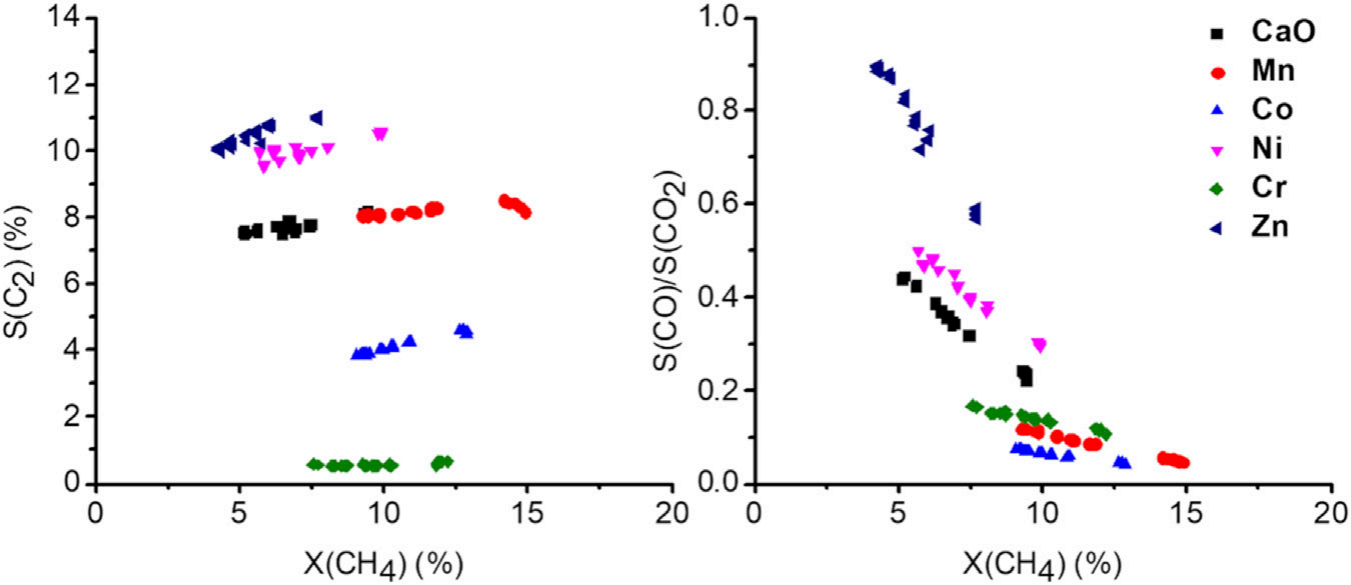
Selectivity-conversion plots at 750°C by adjusting the total gas flow rate (from 37.5 ml/min up to 150 ml/min, 50 mg carbonate precursor, 750 mg SiC, CH_4_:N_2_: O_2_ = 3:3:1).

In Figure 7, selectivity-conversion plots are presented, showing the significantly altered catalytic behavior of the doped catalyst at similar conversions. The effect of flow variation on the C_2+_ selectivity is almost negligible at most a small increase can be observed with increasing conversion, which is mainly due to the increased formation of ethylene (Supplementary Figure S15). The small effect of the conversion on the C_2_ selectivity implies the deep oxidation being a parallel reaction, rather than oxidation of the C_2_ products. With increasing conversion, the CO to CO_2_ ratio drops, indicating CO_2_ being a consecutive product of CO. It has to be considered though, that the contact time variations were conducted varying the volume flows of the gases, rather than changing the catalyst mass. Therefore, other effects might alter the obtained results. In the following paragraph they will be discussed for excluding them from the further discussion.

Due to the increased volume flow, the diffusion film of the heterogeneous catalyst will decrease, and the reaction rate is expected to rise. Recently it was also suggested, that the diffusion barrier might influence the reaction selectivity (Parishan et al., 2018). Another issue that has to be considered is the total increase in conversion, which, due to the exothermic nature of the reaction, will also increase the temperature in the reactor when the flow is increased and the conversion does not decrease by the same factor. Those two effects combined explain why even a decrease of the contact time by a factor of four only results in the reduction of conversion by a factor of 2. Considering the effect of temperature, the highest temperature would be expected at the lowest conversion (4 times the flow, about half conversion resulting in double the amount of heat produced). Looking at the temperature dependence of the reaction (Supplementary Figure S14), the C_2_ selectivity is expected to be increased and CO and CO_2_ selectivity decreased with increasing temperature. As a consequence, the measurements overestimate the C_2_ selectivity at low conversion, which would mean the slight increase observed might, in reality, be more pronounced. The effect of the diffusion film on the selectivity suggests a jump in selectivity upon meeting certain flow conditions (Parishan et al., 2018), which was not observed, instead of steady curves are observed for all products (Supplementary Figure S15). Therefore, an alteration of the obtained results by such an effect can also be excluded.

Comparing now the performance of the doped catalysts, under the before discussed limitation, three groups can be identified. Ni and Zn doping show an increased selectivity towards C_2+_ while in terms of conversion, the effect is not significant. Co and Cr doping lead to a decrease in selectivity, with Cr almost completely preventing any C_2_ products to be formed. In the case of Mn, the S-X trend of CaO appears to be continued, indicating an increase in the abundance of the same active sites. The decrease in selectivity in the case of the Cr and Co-doped sample might be an indication of phase separation, which, however, due to the very low abundance could not be proven or disproven.

For a better understanding of the impact of the dopant on the catalytic performance, the doping level was optimized and a series of Mn and Ni-doped catalysts were synthesized and tested in OCM with dopant concentrations between 0.02 and 0.7 atom% loadings. Since the results for Mn doping and Ni doping yielded similar trends, the results for the Ni doping series are reported in the supporting information (Supplementary Figures S17,18).

**FIGURE 8:**
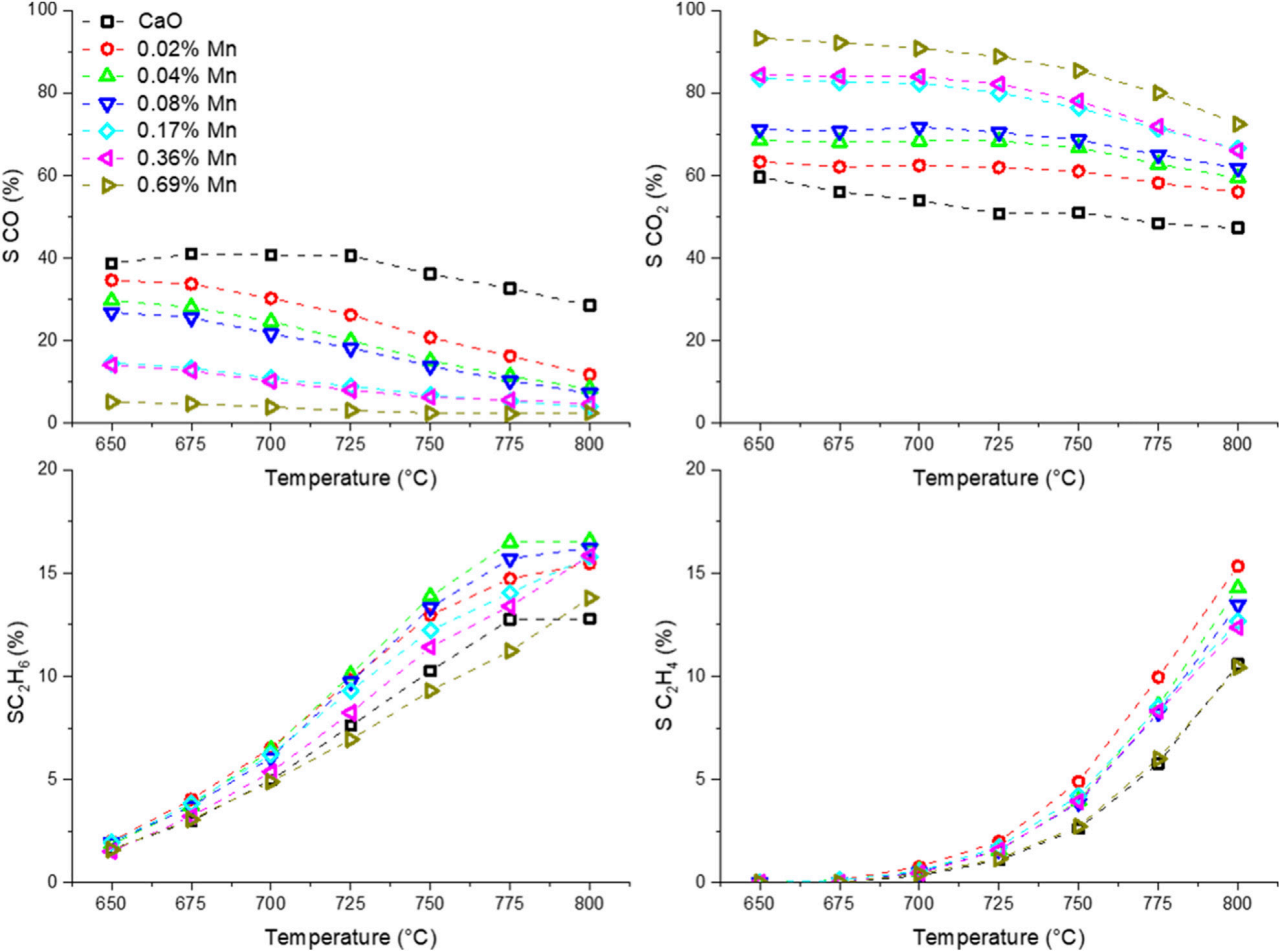
Temperature dependence of OCM products using Mn-doped CaO catalysts (50 mg carbonate precursor, 750 mg SiC, 50 ml/min, CH_4_:N_2_:O_2_ = 3:3:1).

**FIGURE 9:**
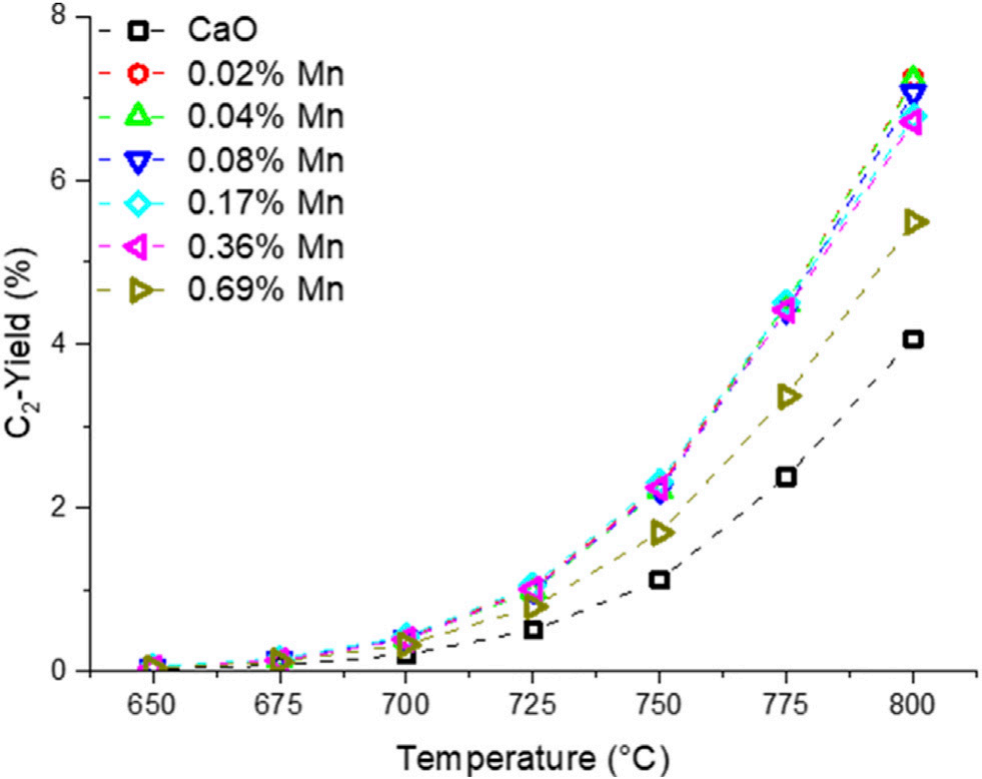
Temperature dependence of combined ethane and ethylene yield of manganese doped CaO catalysts (50 mg carbonate precursor, 750 mg SiC, 50 ml/min, CH_4_:N_2_:O_2_ = 3:3:1).

Before the testing, the catalysts were kept on stream for 3 days at 780 and 800 °C to accelerate the sintering of the catalyst and ensure a stable performance at lower temperatures. In Figure 8, the temperature dependence of the OCM products is presented. The selectivity trend for all the samples is in accordance with the findings made with the differently doped CaO catalysts. A clear trend for the Mn-doped samples can be found for the CO and CO_2_ selectivity. While pure CaO produces significant amounts of CO, the selectivity towards CO decreases with the Mn loading and the CO_2_ selectivity rises accordingly. The individual trends for ethane and ethylene selectivity are less clear. However, the combined ethane and ethylene yield (Figure 9) is similarly enhanced for all but the sample with the highest doping level for which the enhancement is about half of that found for the other doped samples. The improvement by adding Mn as a dopant is quite significant, increasing the yield by 75% (at 800°C), though one has to add, that the overall yield is still pretty low, which might be due to the reaction parameters which were chosen for better comparability and not for best performance. A similar observation could be made in the Ni series (Supplementary Figure S18).

**FIGURE 10:**
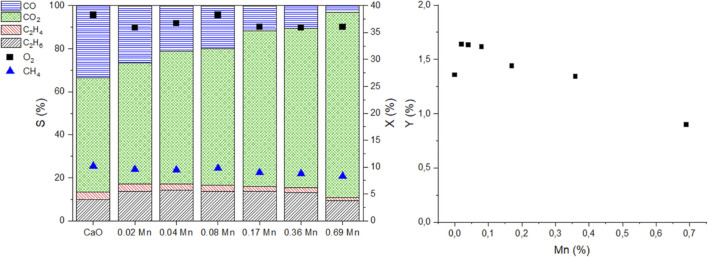
Comparison of catalytic performance of Mn-doped CaO catalysts at 750°C at similar O_2_ conversions (left graph, left axis selectivities of products, right axis CH_4_ and O_2_ conversion), respective C_2_ yield vs. the Mn content (right, 50 mg carbonate precursor, 750 mg SiC, GHSV can be different by a factor of 5).

In Figure 10 the doped catalysts are compared at similar oxygen and methane conversions at 750°C. The catalytic data was again obtained by variation of the volume flow and has to be interpreted under the same restrictions as mentioned before for the metal series data. The full S-X data can be found in the supporting information (Supplementary Figure S16). To achieve similar conversions the contact time between different data points might differ by a factor of 5 (CaO vs 0.69 atom% Mn-doped CaO). For the ethane and ethylene selectivity, a maximum can be found for lower doping concentrations (0.02-0.1 atom%), while with higher doping concentrations, the selectivity declines again. It becomes quite evident that adding Mn promotes the deep oxidation to CO_2_ and strongly reduces the CO selectivity, almost reducing the selectivity to 0 at the highest investigated doping concentration (0.69 atom%). The trend for the deep oxidation products is again different compared to the trend for ethane and ethylene formation.

**FIGURE 11:**
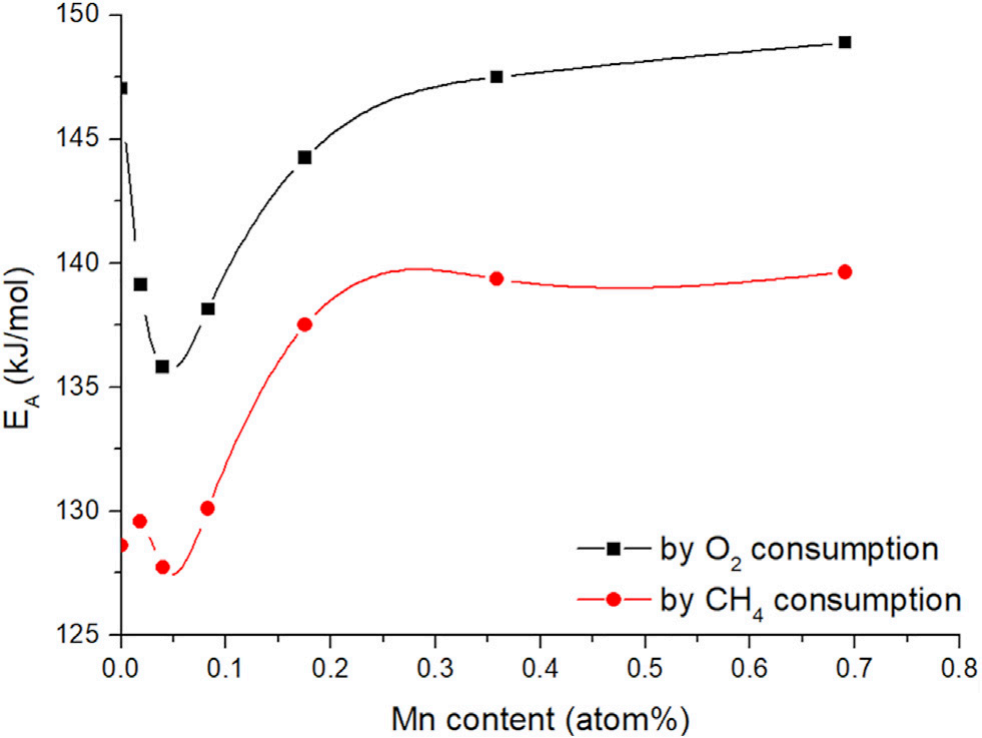
Calculated apparent activation energy of methane and oxygen consumption vs metal content of the Mn (CO, CO_2_, C_2_H_4_, C_2_H_6_) doped CaO catalysts (50 mg carbonate precursor, 750 mg SiC, 50 ml/min 3:3:1 CH_4_:N_2_:O_2_, the temperature range between 650 and 800°C), lines are guides the eye.

Figure 11 (Supplementary Figure S21 for Ni-doped samples) shows the apparent activation energies for oxygen and methane consumption rates as a function of the metal loading concentration obtained by ICP-OES analysis (Arrhenius plots can be viewed in Supplementary Figure S20).

The shape of the trends for both activation energies are quite similar but appear to have an offset of about 15 kJ/mol, which makes sense, looking at the selectivity trends (Figure 8). With increasing temperature, the selectivity towards ethane and ethylene increases, where the selectivity to CO and CO_2_ decreases. Due to deep oxidation consuming more oxygen and less methane compared to coupling and dehydrogenation (Eq. 3 and Eq. 4), the slopes for the individual consumption rates are expected to be different and therefore result in an offset of the activation energy.

$$CH_{4}+\frac{3}{2}O_{2}\to CO+H_{2}O$$ (3)

$$CH_{4}+2O_{2}\to CO_{2}+2H_{2}O$$ (4)

$$2CH_{4}+\frac{1}{2}O_{2}\to C_{2}H_{6}+H_{2}O$$ (5)

$$2CH_{4}+O_{2}\to C_{2}H_{4}+2H_{2}O$$ (6)

It also has to be noted, that the activation energies found in the doping concentration screening are higher compared to those found before, during metal screening (Table 1). This can be explained by the altered synthesis method. Allowing a longer time for the aging of the catalysts (24 vs. 1 h) results in very low surface area precursors (Table 2). It was shown before, that the surface structure of alkaline earth oxides can have a strong impact on the catalytic performance (Schwach et al., 2015b). Nevertheless, even with increased activation energy compared to the previous results, the trend for the activation energies is quite interesting. With small amounts of dopant, the activation barrier is lowered to a certain extent. But, exceeding a dopant concentration of 0.1 atom%, the apparent activation energies for the OCM reaction increase again and level out at similar activation energies as for pure CaO (in case of oxygen consumption rate). It also has to be noted, that the offset between oxygen and methane activation barrier on pure CaO is higher compared to that of doped CaO, indicating that the dopant facilitates the oxygen activation.

## Discussion

Phase pure metal-doped calcium carbonates as oxide precursors were synthesized and no indication for phase separation of the dopants was observed using XRD analysis, neither in the precursors nor in oxide materials, however, this cannot be excluded due to the low total abundance of metal dopants. The doping of metal in the case of Ni appears to have an influence on the crystallization of calcium carbonate during the precipitation process, prolonging the lifetime of the metastable vaterite phase during aging. Whether the Ni^2+^ ion is directly exchanged against a Ca^2+^ in the host lattice, however, remains unclear. In the case of Mn-doped CaO, the EPR measurements could show, that Mn^2+^ occupies high symmetry Ca sites (see also Supplementary Figure S4). Following the Hume-Rothery rule, none of the dopants is likely to form a stable solid solution, due to the significant difference (>15%) in atomic dimension. Due to their low concentration in the host lattice, verification is also very challenging and remains to be shown. In the case of low Fe^3+^ doped CaO, a clustering of the transition metal is known to occur (Biasi and Grillo, 2003), whereas in the case of Mn-doped CaO this appears not to be the case, as indicated by the EPR measurements (Biasi and Grillo, 1999) and also by the lattice contraction observed with XRD.

The addition of transition metal dopants to CaO has a significant influence on the photoluminescence of the sample, which, by oxygen quenching, could be proven to exclusively arise from surface sites. While alkaline dopants have been found to enhance the luminescence intensity of CaO and Ce^3+^ can be used to shift the emission spectrum (Hao et al., 2013), the doping of CaO with transition metals mainly results in a loss of luminescence. Already with the addition of small amounts (0.04 atom% Mn), the overall photoluminescence is strongly reduced, and the luminescence caused by edge sites becomes more prominent (feature at 290 nm, Figure 4 left) due to site isolation (Garrone et al., 1980). The introduced transition metals could offer new energy states favoring the non-radiative recombination for surface excitons. The observed behavior is independent of the nature of the dopant (Supplementary Figure S9) and thus cannot be used to explain the altered selectivities in OCM performance. However, the significant decrease in luminescence intensity confirms the change in the electronic structure of the material surfaces that accompanies the marked change in catalytic activity.

**FIGURE 12:**
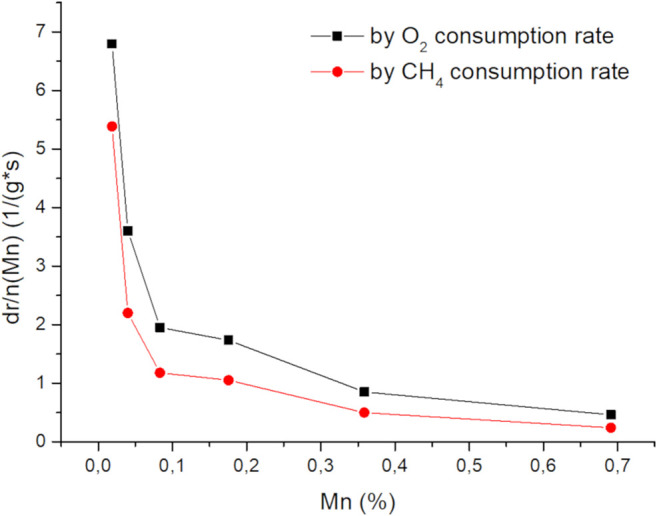
Change of oxygen and methane reaction rates normalized to amount of doping atoms vs total loading (50 mg carbonate precursor, 750 mg SiC, 50 ml/min 3:3:1 CH_4_:N_2_:O_2_, 705°C).

Doping of CaO with Ni and Mn changes the catalytic performance and its spectroscopic properties in a similar way. While low dopant concentrations have a strong impact on both phenomena the doping effect gets less pronounced with increased loading. In Figure 12 (Supplementary Figure S22 for Ni), the change of the reaction rate with the metal loading vs. the metal loading (in relation to the rate of the pure CaO) is plotted, showing that the scarcest dopants have the most significant effect on the reaction rate. Increasing dopant concentrations cause strong PL quenching and significant signal broadening in EPR, indicating changes in the interactions with the adsorbed oxygen as well as among the dopants themselves. With increasing dopant concentrations, the average distance between two dopant atoms decreases down to the range of dipole exchange interactions which is known for Mn^2+^ to be about 0.59 nm, which is well in the region of the here applied doping amounts and might be an explanation for the observed behavior (Biasi and Grillo, 1999). Further decreases of the distance could cause clustering of the metal atoms leading to a cancellation of the doping effect, indicated by the again increasing activation energies. For other metals this distance can be even larger, e.g., 0.83 nm for Gd^3+^, and 0.68 nm for Cu^2+^, where in the case of Fe, it is known for clustering at distances below a similar range (Biasi and Grillo, 2002a; Biasi and Grillo, 2002b; Biasi and Grillo, 2003).

Whether these observations support the model postulated by Freund et al., meaning a dopant-adsorbent interaction (Nilius and Freund, 2015), remains unclear. Doping aimed to alter the electronic structure should result in a significantly changed photoluminescence spectrum, such as significant shifts, or the creation of bulk new phosphors (Hao et al., 2013). However, proving with oxygen quenching, that the PL only derives from the material surface, the only observation made was a change in intensity, as well as a shift of the intensity distributions already existing in CaO, indicating a change in the surface structure. The observed effect can also not be explained by the “healing” of defects on the CaO surface. Defect sites, in general, are more reactive compared to pristine surfaces, which in the case of CaO is even postulated to be inactive (Nilius and Freund, 2015; Aljama et al., 2017, Aljama et al., 2018). The healing of defects should mainly result in an altered selectivity, as well as reduced activity, which is not observed in contrast, activity, as well as selectivity, are altered simultaneously. A more compelling explanation for the altered catalytic activity could therefore be the change of the crystallization due to the presence of foreign atoms with a significantly different ionic radius. An indication of this can be seen in the X-S plots (Figure 7). The Mn series appears to continue the C2-selectivity trend of the undoped CaO, implying the presence of more of the same active sites. As the PL measurements suggested, the ratio of the lower coordinated site compared to higher coordinated sites is higher in the doped samples. With highly coordinated sites, such as terrace sites being considered inactive in the OCM reaction, the increase of the lower coordinated sites explains the observed higher activity of the Mn-doped CaO. It is also quite reasonable that this effect is limited since highly isolated foreign ions can have a large impact on the surface structure by introducing lattice distortions, strain, stress (Khranovskyy et al., 2014), when the foreign atoms are getting close to each other, shown by the signal broadening in the EPR spectra (Figure 6B), the effect might be canceled out. Seeing a similar behavior with the Ni and Mn series also supports this hypothesis. A definitive proof, however, can unfortunately not be provided and more research is needed. With this, the initial research questions can be answered.

In the case of Mn doping, EPR experiments could verify Mn^2+^ in low concentrations positioned in high symmetry sites, most likely to substitute Ca^2+^ ions in the lattice.

No indication could be found for the dopants to introduce new energy states; however, their presence significantly alters the surface structure of the catalyst, indicated by the PL.

In the case of Mn doping, the post-reaction EPR measurements showed no indication for a change of the oxidation state of Mn, The catalyst selectivity can be enhanced via transition metal doping, with not all chosen elements being suitable. Especially ions with a stable 2+ oxidation state can enhance the catalyst selectivity if introduced in quantities up to 0.1 atom%.

## Conclusion

Phase pure precursor materials could be synthesized by a co-precipitation method while avoiding disturbing contaminants such as halides and alkalines. Upon activation to CaO, no indications for phase segregation could be observed with the applied methods. Mn dopants probed by EPR exhibit O_h_ site symmetry as expected for substitutional insertion and no indication for clustering was found for Mn concentrations up to 0.36 mol%. In catalytic tests, metal dopants with a preferential oxidation state of two were found to have a beneficial effect on the OCM reaction (Mn, Ni, Zn), whereas other dopants, promoting the formation of cationic vacancies, were found to be unfavorable (Co, Cr) leading to increased methane combustion. Synthesizing a series of Mn and Ni-doped CaO, a minimum in the activation energy and was found at doping concentrations around ∼0.04%. Increasing the amounts of doping results in a reduction of efficiency of the individual doping atom. These findings are in line with observations made in PL and EPR, showing that increased doping results in loss of intensity (PL) and signal broadening (EPR), which indicates a dopant-dopant interaction not beneficial for the catalyst optimization. In conclusion, transition metal doping in low quantities can be applied to improve the catalytic performance of CaO, but it has to be noted, that the overall effect, in this case, is limited.

## Data Availability

The raw data supporting the conclusion of this article will be made available by the authors, without undue reservation.

## References

[B1] Abrams B. L. Holloway P. H. (2004). Role of the Surface in Luminescent Processes. Chem. Rev. 104, 5783–5802. 10.1021/cr020351r 15584688

[B2] Aljama H. Nørskov J. K. Abild-Pedersen F. (2017). Theoretical Insights into Methane C-H Bond Activation on Alkaline Metal Oxides. J. Phys. Chem. C 121, 16440–16446. 10.1021/acs.jpcc.7b05838

[B3] Aljama H. Nørskov J. K. Abild-Pedersen F. (2018). Tuning Methane Activation Chemistry on Alkaline Earth Metal Oxides by Doping. J. Phys. Chem. C 122, 22544–22548. 10.1021/acs.jpcc.8b06682

[B4] Arndt S. Laugel G. Levchenko S. Horn R. Baerns M. Scheffler M. (2011). A Critical Assessment of Li/MgO-Based Catalysts for the Oxidative Coupling of Methane. Catal. Rev. 53, 424–514. 10.1080/01614940.2011.613330

[B5] Bajdich M. Nørskov J. K. Vojvodic A. (2015). Surface Energetics of Alkaline-Earth Metal Oxides: Trends in Stability and Adsorption of Small Molecules. Phys. Rev. B 91, 1–10. 10.1103/PhysRevB.91.155401

[B6] Beck B. Harth M. Hamilton N. G. Carrero C. Uhlrich J. J. Trunschke A. (2012). Partial Oxidation of Ethanol on Vanadia Catalysts on Supporting Oxides with Different Redox Properties Compared to Propane. J. Catal. 296, 120–131. 10.1016/j.jcat.2012.09.008

[B7] Bengoechea-Encabo A. Albert S. Sánchez-Garcia M. A. Calleja E. (2016). Oxygen-related Photoluminescence Quenching in Selectively Grown GaN Nanocolumns: Dependence on Diameter. Mater. Sci. Semiconductor Process. 55, 59–62. 10.1016/j.mssp.2016.03.018

[B8] Borgwardt R. H. (1989). Calcium Oxide Sintering in Atmospheres Containing Water and Carbon Dioxide. Ind. Eng. Chem. Res. 28, 493–500. 10.1021/ie00088a019

[B9] Cavenett B. C. (1964). The Allowed and Forbidden Transitions in the Paramagnetic Resonance of the Manganese Ion in Cubic Zinc Selenide. Proc. Phys. Soc. 84, 1–9. 10.1088/0370-1328/84/1/302

[B10] Coluccia S. Deane A. M. Tench A. J. (1978). Photoluminescent Spectra of Surface States in Alkaline Earth Oxides. J. Chem. Soc. Faraday Trans. 1 74, 2913–2922. 10.1039/f19787402913

[B11] de Biasi R. S. Grillo M. L. N. (2003). Evidence for Clustering in Fe^3+^-Doped CaO. J. Phys. Chem. Sol. 64, 711–713. 10.1016/s0022-3697(02)00371-2

[B12] de Biasi R. S. Grillo M. L. N. (2002a). Influence of Copper Concentration on the ESR Spectrum of Cu^2+^ in CaO. Solid State. Commun. 121, 697–700. 10.1016/s0038-1098(01)00519-1

[B13] de Biasi R. S. Grillo M. L. N. (2002b). Influence of Gadolinium Concentration on the ESR Spectrum of Gd^3+^ in CaO. Solid State. Commun. 124, 131–133. 10.1016/s0038-1098(02)00479-9

[B14] de Biasi R. S. Grillo M. L. N. (1999). Influence of Manganese Concentration on the ESR Spectrum of Mn^2+^ in CaO. J. Alloys Comp. 282, 5–7. 10.1016/s0925-8388(98)00701-4

[B15] Elkins T. W. Roberts S. J. Hagelin-Weaver H. E. (2016). Effects of Alkali and Alkaline-Earth Metal Dopants on Magnesium Oxide Supported Rare-Earth Oxide Catalysts in the Oxidative Coupling of Methane. Appl. Catal. A: Gen. 528, 175–190. 10.1016/j.apcata.2016.09.011

[B16] Garrone E. Zecchina A. Stone F. S. (1980). An Experimental and Theoretical Evaluation of Surface States in MgO and Other Alkaline Earth Oxides. Philosophical Mag. B 42, 683–703. 10.1080/01418638008224034

[B17] Haber J. Witko M. (2003). Oxidation Catalysis-Electronic Theory Revisited. J. Catal. 216, 416–424. 10.1016/S0021-9517(02)00037-4

[B18] Hao Z. Zhang X. Luo Y. Zhang L. Zhao H. Zhang J. (2013). Enhanced Ce^3+^ Photoluminescence by Li^+^ Co-doping in CaO Phosphor and its Use in Blue-Pumped white LEDs. J. Lumin. 140, 78–81. 10.1016/j.jlumin.2013.03.013

[B19] Hermans I. Spier E. S. Neuenschwander U. Turrà N. Baiker A. (2009). Selective Oxidation Catalysis: Opportunities and Challenges. Top. Catal. 52, 1162–1174. 10.1007/s11244-009-9268-3

[B20] Holleman A. F. Wiberg N. Wiberg E. (2007). “Kapitel XVII Die Gruppe der Erdalkalimetalle,” Lehrbuch der Anorganischen Chemie. Berlin: Walter de Gruyter, 1215–1258. 10.1515/9783110206845

[B21] Hu Z. Li B. Sun X. Metiu H. (2011). Chemistry of Doped Oxides: The Activation of Surface Oxygen and the Chemical Compensation Effect. J. Phys. Chem. C 115, 3065–3074. 10.1021/jp110333z

[B22] Ito T. Wang J. Lin C. H. Lunsford J. H. (1985). Oxidative Dimerization of Methane over a Lithium-Promoted Magnesium Oxide Catalyst. J. Am. Chem. Soc. 107, 5062–5068. 10.1021/ja00304a008

[B23] Khranovskyy V. Tsiaoussis I. Eriksson M. Yakimova R. (2014). Effect of Ag Doping on the Microstructure and Photoluminescence of ZnO Nanostructures. Phys. Status Solidi A. 211, 2109–2114. 10.1002/pssa.201400008

[B24] Kondratenko E. V. Wolf D. Baerns M. (1999). Influence of Electronic Properties of Na_2_O/CaO Catalysts on Their Catalytic Characteristics for the Oxidative Coupling of Methane. Catal. Lett. 58, 217–223. 10.1023/A:1019058724099

[B25] Lee J. S. Oyama S. T. (1988). Oxidative Coupling of Methane to Higher Hydrocarbons. Catal. Rev. 30, 249–280. 10.1080/01614948808078620

[B26] Lin C. Wang J. X. Lunsford J. H. (1988). Oxidative Dimerization of Methane over Sodium-Promoted Calcium Oxide. J. Catal. 111, 302–316. 10.1016/0021-9517(88)90089-9

[B27] MacLean S. G. Duley W. W. (1984). Photoluminescence from Surface States in MgO and CaO Powders. J. Phys. Chem. Sol. 45, 227–235. 10.1016/0022-3697(84)90123-9

[B28] McFarland E. W. Metiu H. (2013). Catalysis by Doped Oxides. Chem. Rev. 113, 4391–4427. 10.1021/cr300418s 23350590

[B29] Müller M. Sternig A. Stankic S. Stöger-Pollach M. Bernardi J. Knözinger E. (2008). Nanoparticles as a Support: CaO Deposits on MgO Cubes. J. Phys. Chem. C 112, 9120–9123. 10.1021/jp802854z

[B30] Nebel H. Epple M. (2008). Continuous Preparation of Calcite, Aragonite and Vaterite, and of Magnesium-Substituted Amorphous Calcium Carbonate (Mg-ACC). Z. Anorg. Allg. Chem. 634, 1439–1443. 10.1002/zaac.200800134

[B31] Nguyen T. N. Nhat T. T. P. Takimoto K. Thakur A. Nishimura S. Ohyama J. (2020). High-Throughput Experimentation and Catalyst Informatics for Oxidative Coupling of Methane. ACS Catal. 10, 921–932. 10.1021/acscatal.9b04293

[B32] Nilius N. Freund H.-J. (2015). Activating Nonreducible Oxides via Doping. Acc. Chem. Res. 48, 1532–1539. 10.1021/acs.accounts.5b00018 25894859

[B33] Ohyama J. Kinoshita T. Funada E. Yoshida H. Machida M. Nishimura S. (2021). Direct Design of Active Catalysts for Low Temperature Oxidative Coupling of Methane via Machine Learning and Data Mining. Catal. Sci. Technol. 11, 524–530. 10.1039/d0cy01751e

[B34] Papa F. Gingasu D. Patron L. Miyazaki A. Balint I. (2010). On the Nature of Active Sites and Catalytic Activity for OCM Reaction of Alkaline-Earth Oxides-Neodymia Catalytic Systems. Appl. Catal. A: Gen. 375, 172–178. 10.1016/j.apcata.2009.12.039

[B35] Parishan S. Nowicka E. Fleischer V. Schulz C. Colmenares M. G. Rosowski F. (2018). Investigation into Consecutive Reactions of Ethane and Ethene under the OCM Reaction Conditions over Mn_x_O_y_-Na_2_WO_4_/SiO_2_ Catalyst. Catal. Lett. 148, 1659–1675. 10.1007/s10562-018-2384-6

[B36] Puust L. Kiisk V. Eltermann M. Mändar H. Saar R. Lange S. (2017). Effect of Ambient Oxygen on the Photoluminescence of Sol-Gel-Derived Nanocrystalline ZrO_2_:Eu,Nb. J. Phys. D: Appl. Phys. 50, 215303. 10.1088/1361-6463/aa6c48

[B37] Rane V. H. Chaudhari S. T. Choudhary V. R. (2006). Comparison of the Surface and Catalytic Properties of Rare Earth-Promoted CaO Catalysts in the Oxidative Coupling of Methane. J. Chem. Technol. Biotechnol. 81, 208–215. 10.1002/jctb.1387

[B38] Rane V. H. Chaudhari S. T. Choudhary V. R. (2008). Influence of Alkali Metal Doping on Surface Properties and Catalytic Activity/Selectivity of CaO Catalysts in Oxidative Coupling of Methane. J. Nat. Gas Chem. 17, 313–320. 10.1016/S1003-9953(09)60001-3

[B39] Schlögl R. (2015). Heterogeneous Catalysis. Angew. Chem. Int. Ed. 54, 3465–3520. 10.1002/anie.201410738 25693734

[B40] Schwach P. Frandsen W. Willinger M.-G. Schlögl R. Trunschke A. (2015a). Structure Sensitivity of the Oxidative Activation of Methane over MgO Model Catalysts: I. Kinetic Study. J. Catal. 329, 560–573. 10.1016/j.jcat.2015.05.007

[B41] Schwach P. Hamilton N. Eichelbaum M. Thum L. Lunkenbein T. Schlögl R. (2015b). Structure Sensitivity of the Oxidative Activation of Methane over MgO Model Catalysts: II. Nature of Active Sites and Reaction Mechanism. J. Catal. 329, 574–587. 10.1016/j.jcat.2015.05.008

[B42] Stankic S. Bernardi J. Diwald O. Knözinger E. (2006). Optical Surface Properties and Morphology of MgO and CaO Nanocrystals. J. Phys. Chem. B 110, 13866–13871. 10.1021/jp061741a 16836335

[B43] Stankic S. Müller M. Diwald O. Sterrer M. Knözinger E. Bernardi J. (2005). Size-Dependent Optical Properties of MgO Nanocubes. Angew. Chem. Int. Ed. 44, 4917–4920. 10.1002/anie.200500663 15999373

[B44] Stoll S. Jeschke G. Willer M. Schweiger A. (1998). Nutation-Frequency Correlated EPR Spectroscopy: The PEANUT Experiment. J. Magn. Reson. 130, 86–96. 10.1006/jmre.1997.1285 9469902

[B45] Sun M. Zhang J. Putaj P. Caps V. Lefebvre F. Pelletier J. (2014). Catalytic Oxidation of Light Alkanes (C1-C4) by Heteropoly Compounds. Chem. Rev. 114, 981–1019. 10.1021/cr300302b 24245654

[B46] Sun X. Li B. Metiu H. (2013). Methane Dissociation on Li-, Na-, K-, and Cu-Doped Flat and Stepped CaO(001). J. Phys. Chem. C 117, 7114–7122. 10.1021/jp4002803

[B47] Thum L. Rudolph M. Schomäcker R. Wang Y. Tarasov A. Trunschke A. (2019). Oxygen Activation in Oxidative Coupling of Methane on Calcium Oxide. J. Phys. Chem. C 123, 8018–8026. 10.1021/acs.jpcc.8b07391

[B48] van Geet A. L. (1968). Ammonia Exchange of Nickel-Ammine Complex in Aqueous Ammonia Measured by Proton Magnetic Resonance. Inorg. Chem. 7, 2026–2029. 10.1021/ic50068a013

[B49] Voskresenskaya E. N. Roguleva V. G. Anshits A. G. (1995). Oxidant Activation over Structural Defects of Oxide Catalysts in Oxidative Methane Coupling. Catal. Rev. 37, 101–143. 10.1080/01614949508007092

[B50] Xiao-Dong W. Tysoe W. T. Greenler R. G. Truszkowska K. (1991). A Reflection-Absorption Infrared Spectroscopy Study of the Adsorption of Atomic Oxygen on Silver. Surf. Sci. 257, 335–343. 10.1016/0039-6028(91)90804-2

[B51] Zavyalova U. Holena M. Schlögl R. Baerns M. (2011). Statistical Analysis of Past Catalytic Data on Oxidative Methane Coupling for New Insights into the Composition of High-Performance Catalysts. ChemCatChem 3, 1935–1947. 10.1002/cctc.201100186

[B52] Zhang Y. Xu J. Xu X. Xi R. Liu Y. Fang X. (2020). Tailoring La_2_Ce_2_O_7_ Catalysts for Low Temperature Oxidative Coupling of Methane by Optimizing the Preparation Methods. Catal. Today 355, 518–528. 10.1016/j.cattod.2019.06.060

[B53] Zhu Y. Wu S. Wang X. (2011). Nano CaO Grain Characteristics and Growth Model under Calcination. Chem. Eng. J. 175, 512–518. 10.1016/j.cej.2011.09.084

