# Supplementary material for: Transition-Metal-Doping of CaO as Catalyst for the OCM Reaction, a Reality Check
Source: Front Chem. 2022 Feb 11;10:768426. doi: 10.3389/fchem.2022.768426 (PMC8876934; doi:10.3389/fchem.2022.768426)
Supplement: Supplementary file 1 [file DataSheet1.pdf]

Supplementary Material

Supplementary Data

1 Synthesis and Characterization

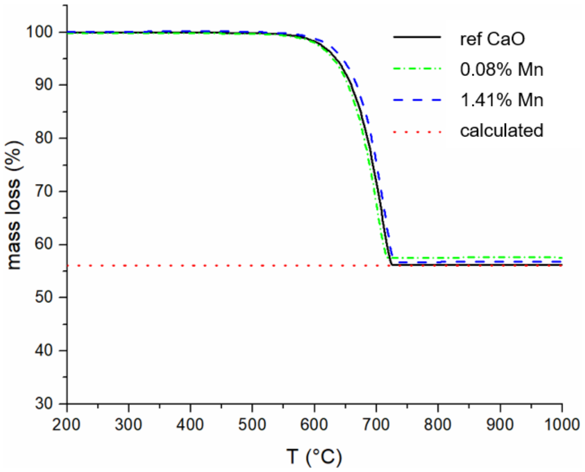

Figure S1: Decomposition of manganese doped $CaCO_3$ under synthetic air in a thermogravimetric balance at 1 °C/min up to 1000 °C

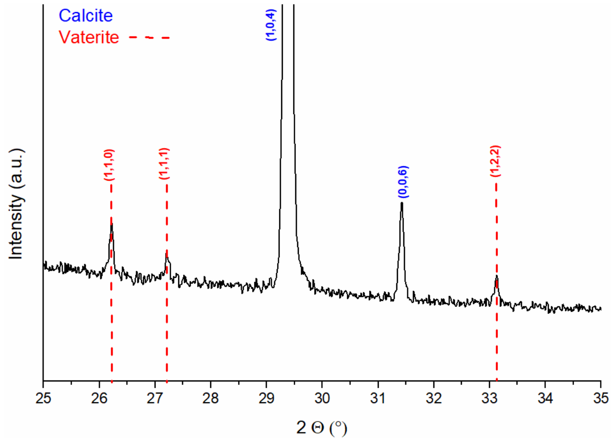

Figure S2: XRD pattern of precipitated 1.60 $NiCaCO_3$.

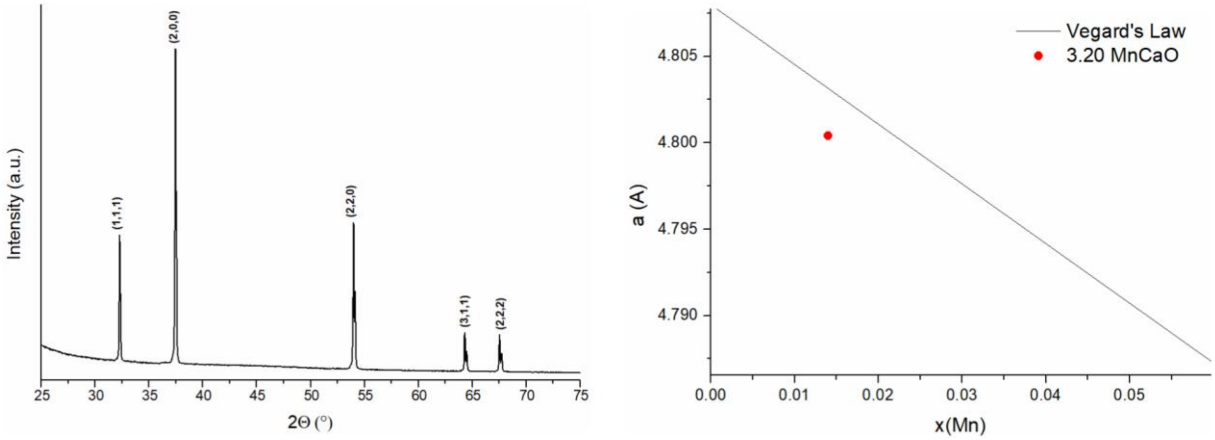

Figure S3: XRD pattern of Mn-doped CaO (left, 1.41 atom% Mn, decomposed for 6 hours at 900 °C applying a heating rate of 5 °C/min), lattice parameter A compared to Vegard's Law assuming solid solution of CaO and MnO.

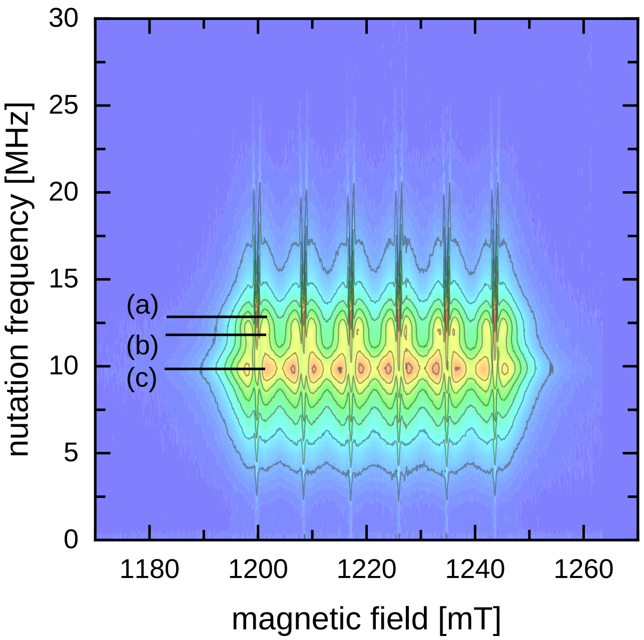

Figure S4: 34 GHz transient nutation EPR measurement of CaO with 0.04 mol% Ca at 20 K for assignment of narrow lines and powder pattern to the (a) │±1/2>↔ │±1/2>, (b) │±3/2>↔│±1/2> and (c) │±5/2>↔│±3/2> transitions in the multilevel spin system. The observed ratio of frequencies is in agreement with the ratio of theoretical values 3, 2.83, 2.23. The experimental value detected for the nutation frequency of the │±5/2>↔│±3/2> transition of 10 MHz is in agreement with the independently observed frequency (12 MHz) of a S =1/2 spin system under full mw power, considering the 9 dB lower mw power used in the current experiment.

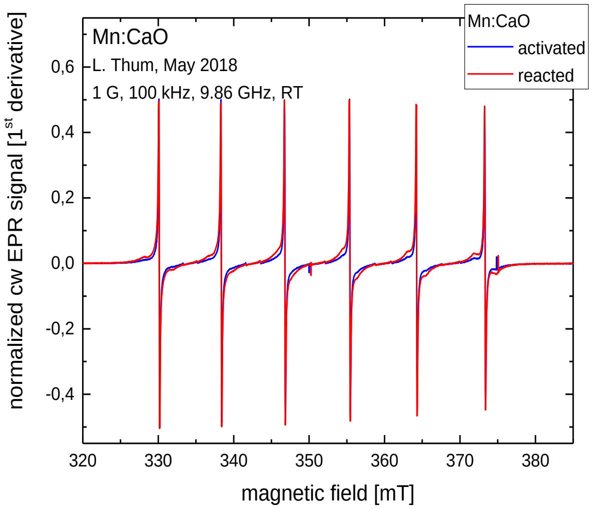

Figure S5: EPR spectra of 0.04%Mn doped sample after activation (900°C for 6h in synthetic air) and after reaction (750°C 4:4:1 CH_4:N_2:O_2 for 12h). Signals almost identical, no change of Mn species during reaction.

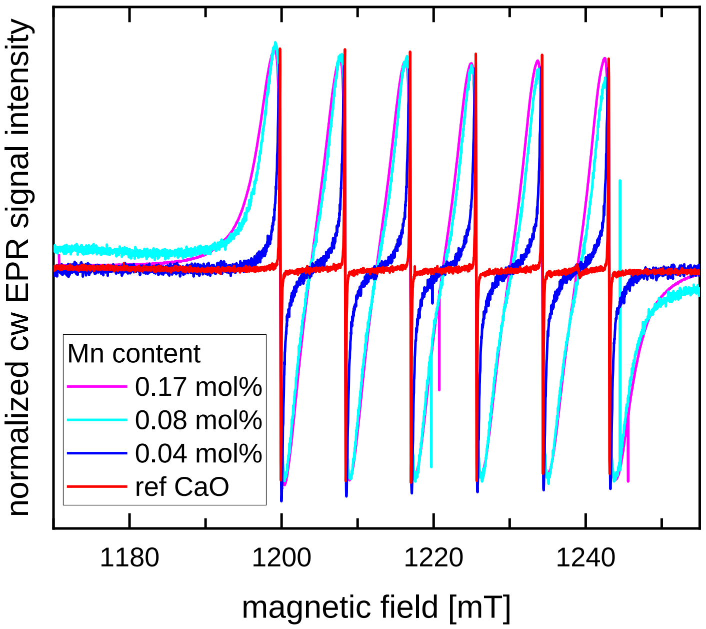

Figure S6: 34 GHz cw EPR spectra of Mn doped CaO measured at room temperature. Shard lines at low concentration indication for isolated Mn species in CaO host lattice. Line broadening indicates enhanced dipol-dipol interaction with increased Mn doping as shown in Figure 6b.

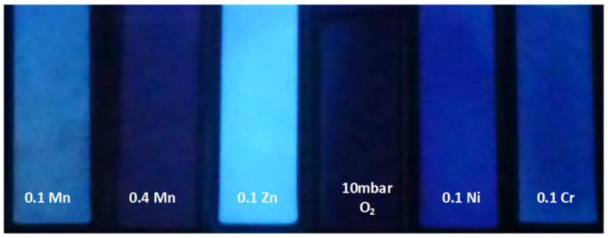

Figure S7: Photograph of doped CaO samples sealed in a vacuum ($p < 5*10^{-6}$ mbar) under UV irradiation (4th sample CaO in 10 mbar O_2 atmosphere).

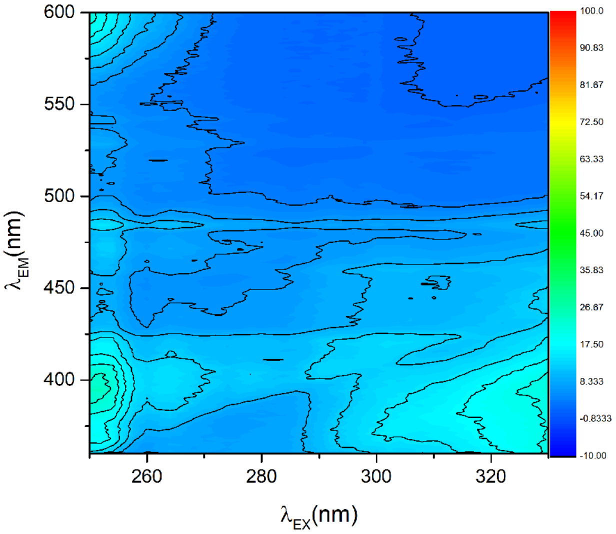

Figure S8: Photoluminescence spectra at room temperature of 1.41% Mn CaO activated at 900°C under dynamic vacuum for 6h.

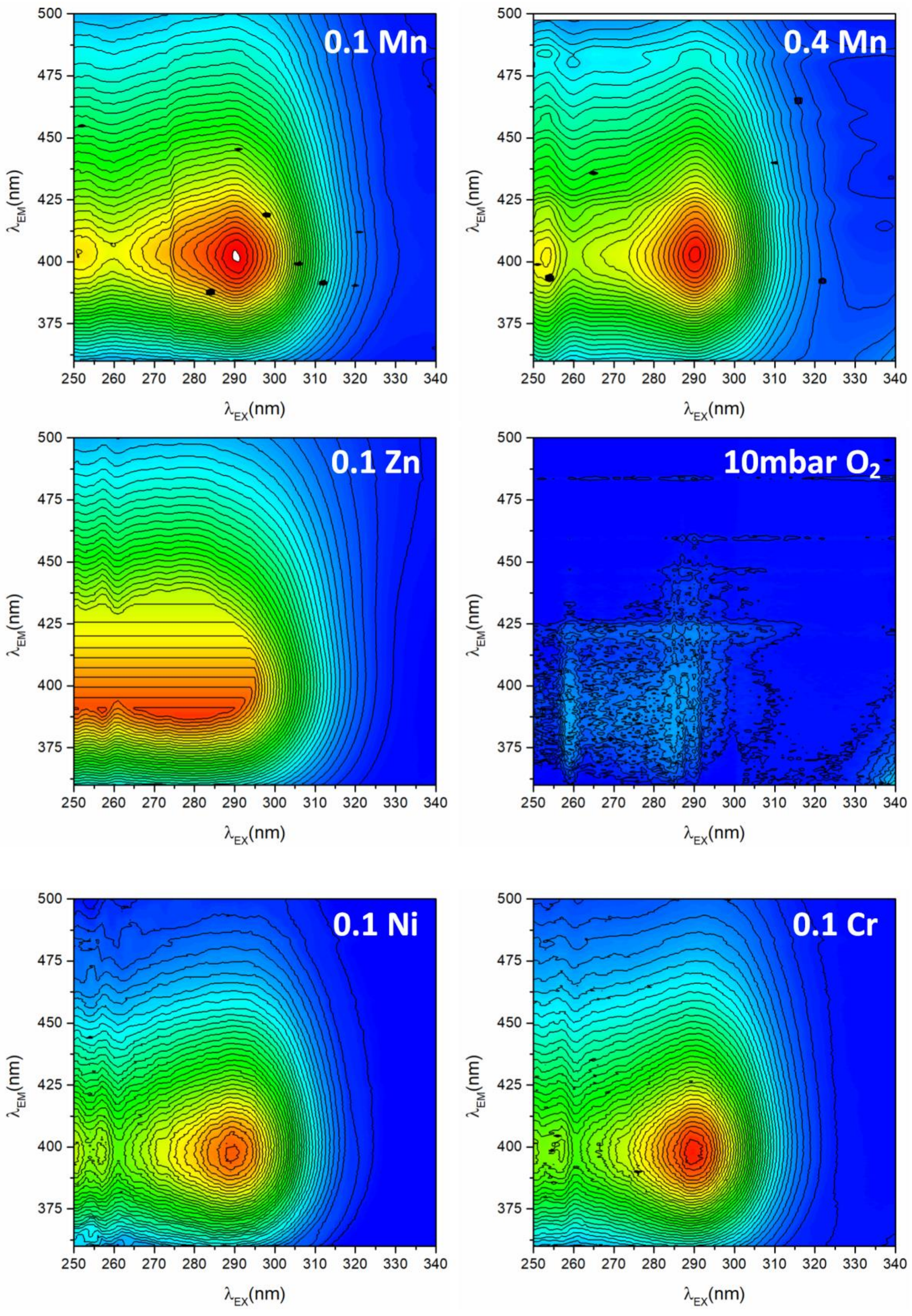

Figure S9: Doped transition metal-doped CaO samples activated at 900°C under dynamic vacuum for 6h and sealed afterwards in the cuvette.

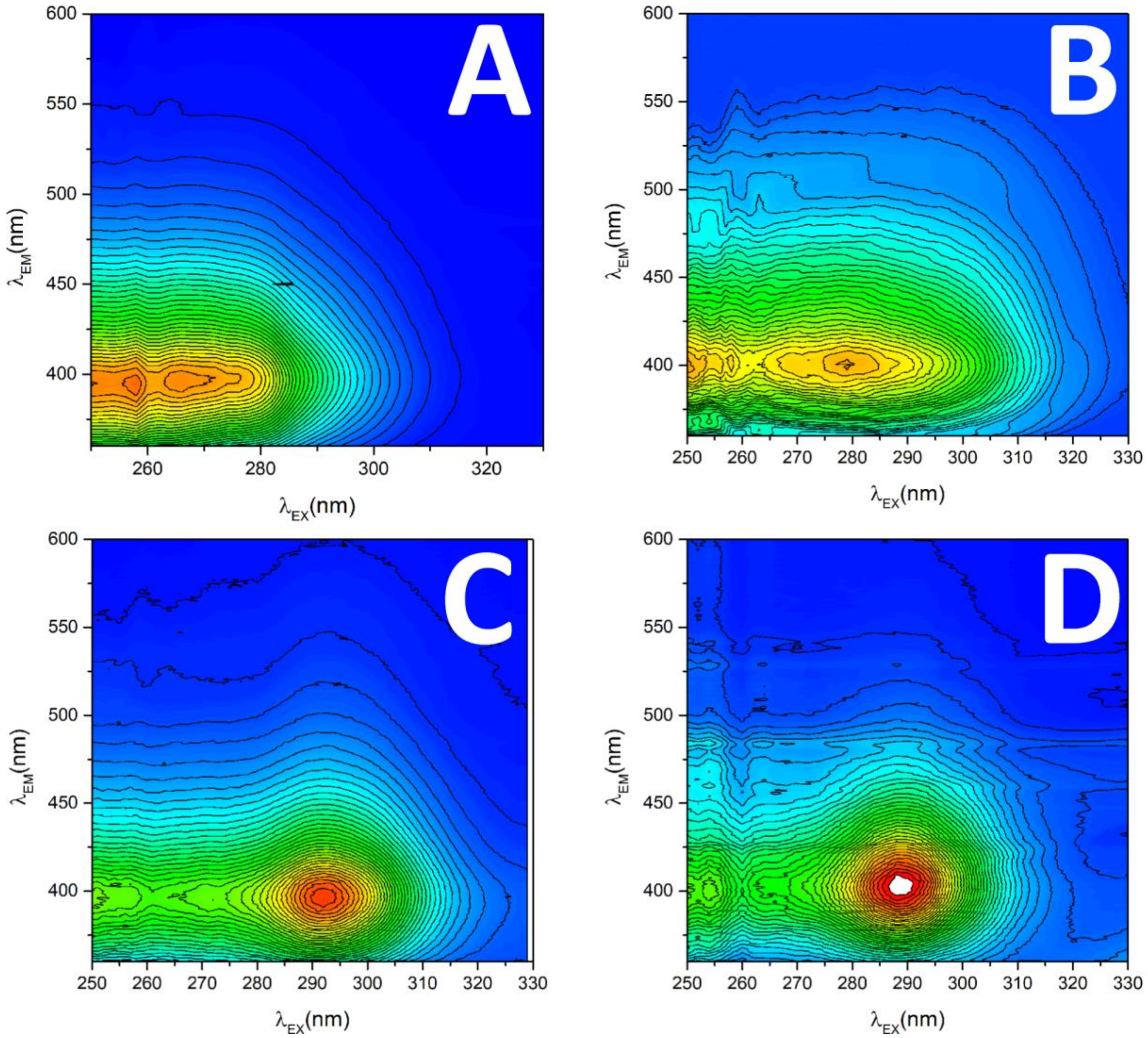

Figure S10: PL spectra of CaO (A: pure CaO, B: ref CaO) and Mn-doped CaO (C: 0.1 MnCaO, D: 0.4 MnCaO activated at 900°C in a vacuum (It has to be noted the overall intensities follows the trend A>B>>C>D).

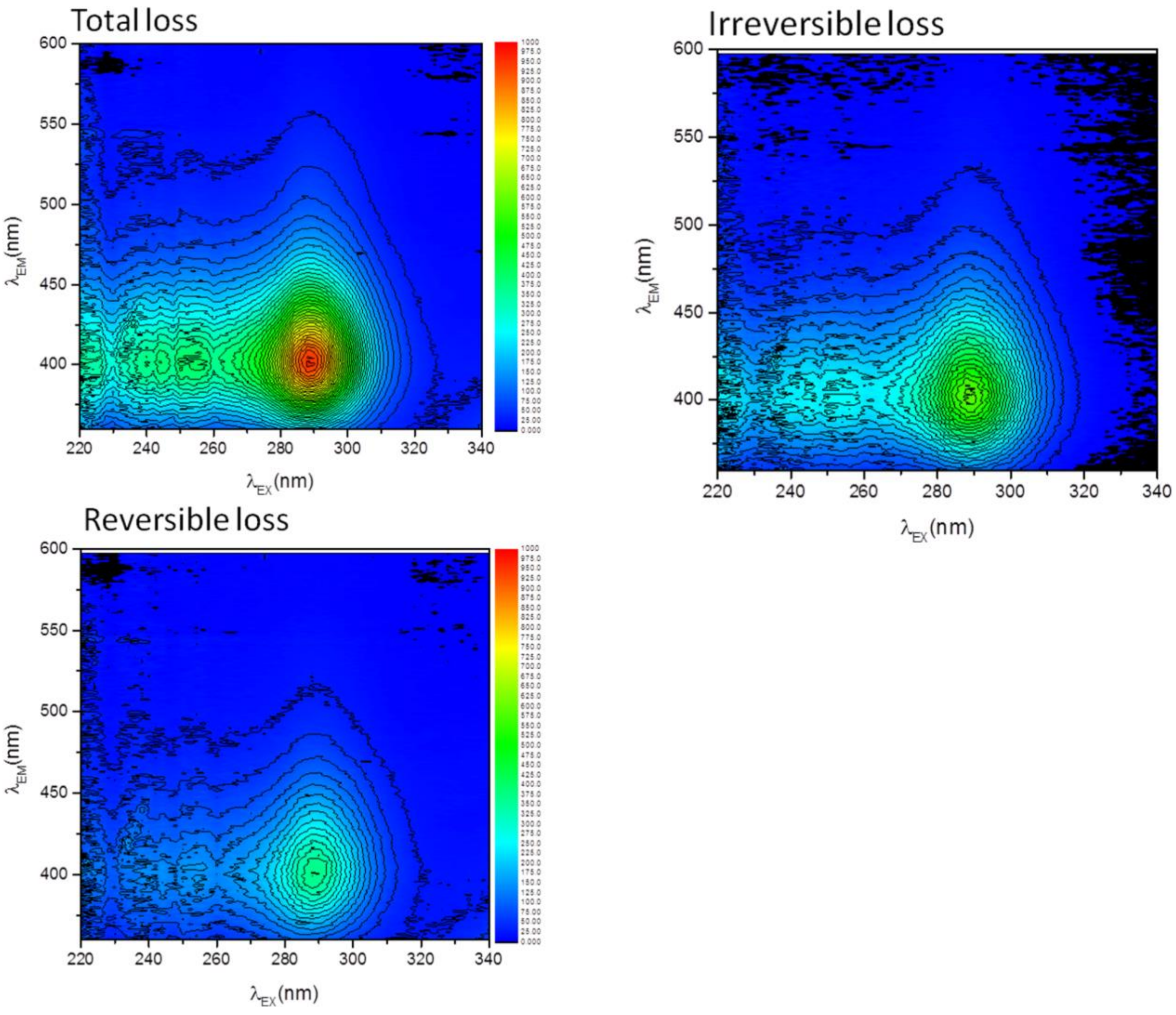

Figure S11: Total, reversible and irreversible loss of photoluminescence signal (measured PL signal minus PL signal obtained in oxygen atmosphere) upon adding 1 mbar O_2 oxygen to Mn-doped CaO (0.4 mn CaO) activated at 900 °C in vacuum.

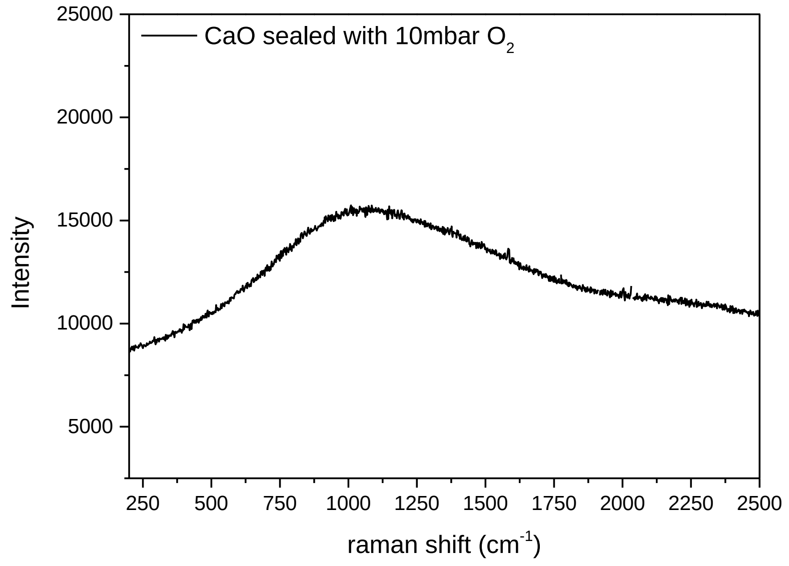

Figure S12: Raman spectra of CaO sealed in a quartz cuvette in 10 mbar oxygen atmosphere at room temperature, a 633 nm laser was used.

2 Additional Catalytic Data

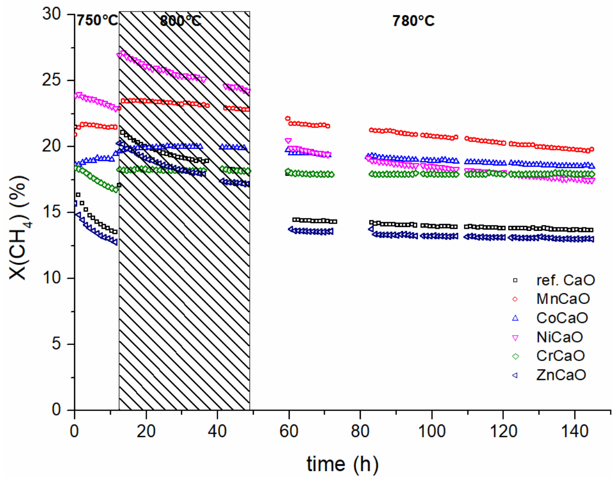

Figure S13: OCM time on stream experiments (50 mg cat, 750 mg SiC, 50 ml/min, 3:3:1, CH_4:N_2:O_2) for metal doped CaO (<0.1mol%).

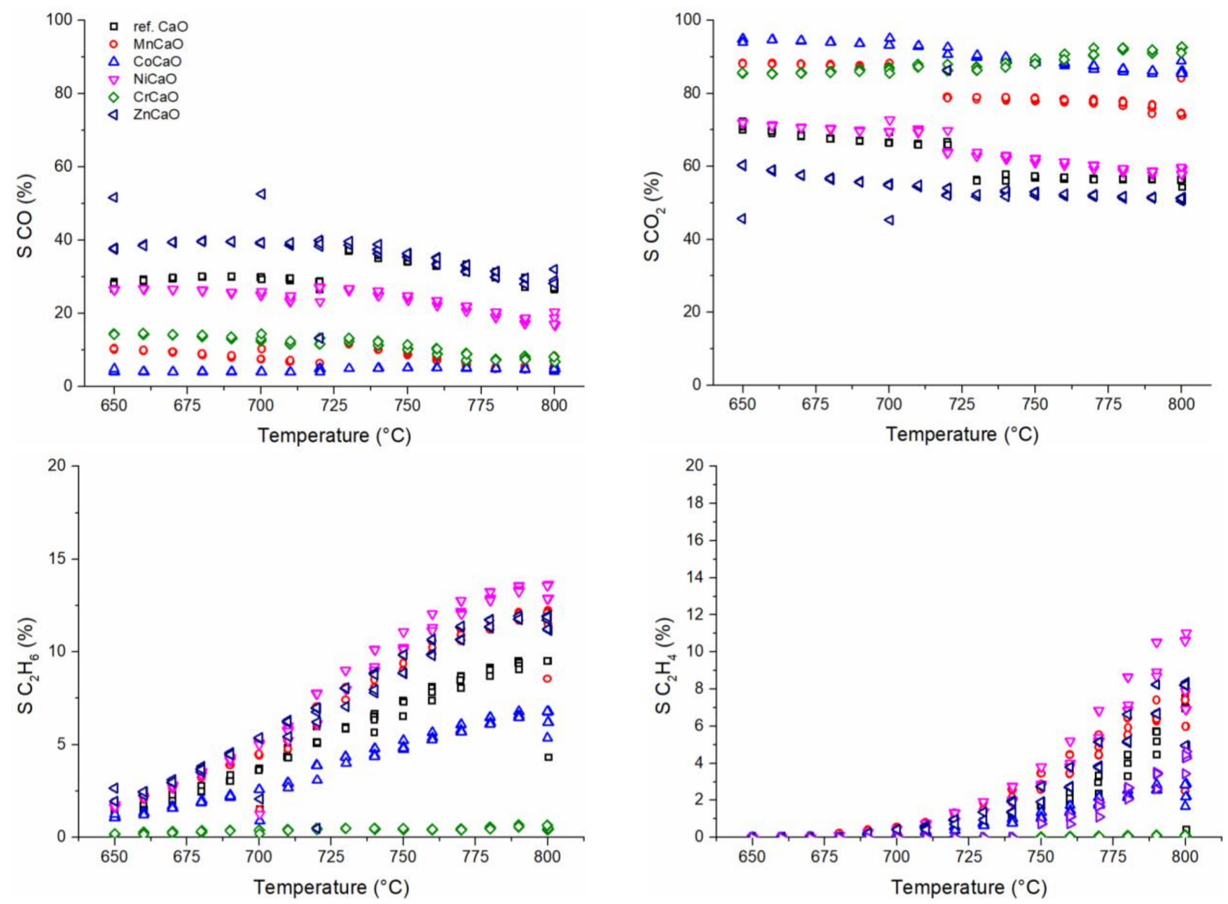

Figure S14: Temperature dependence of OCM products using doped CaO catalysts (metal content <0.1%, 50 mg carbonate precursor, 750 mg SiC, 50 ml/min 3:3:1 CH_4:N_2:O_2).

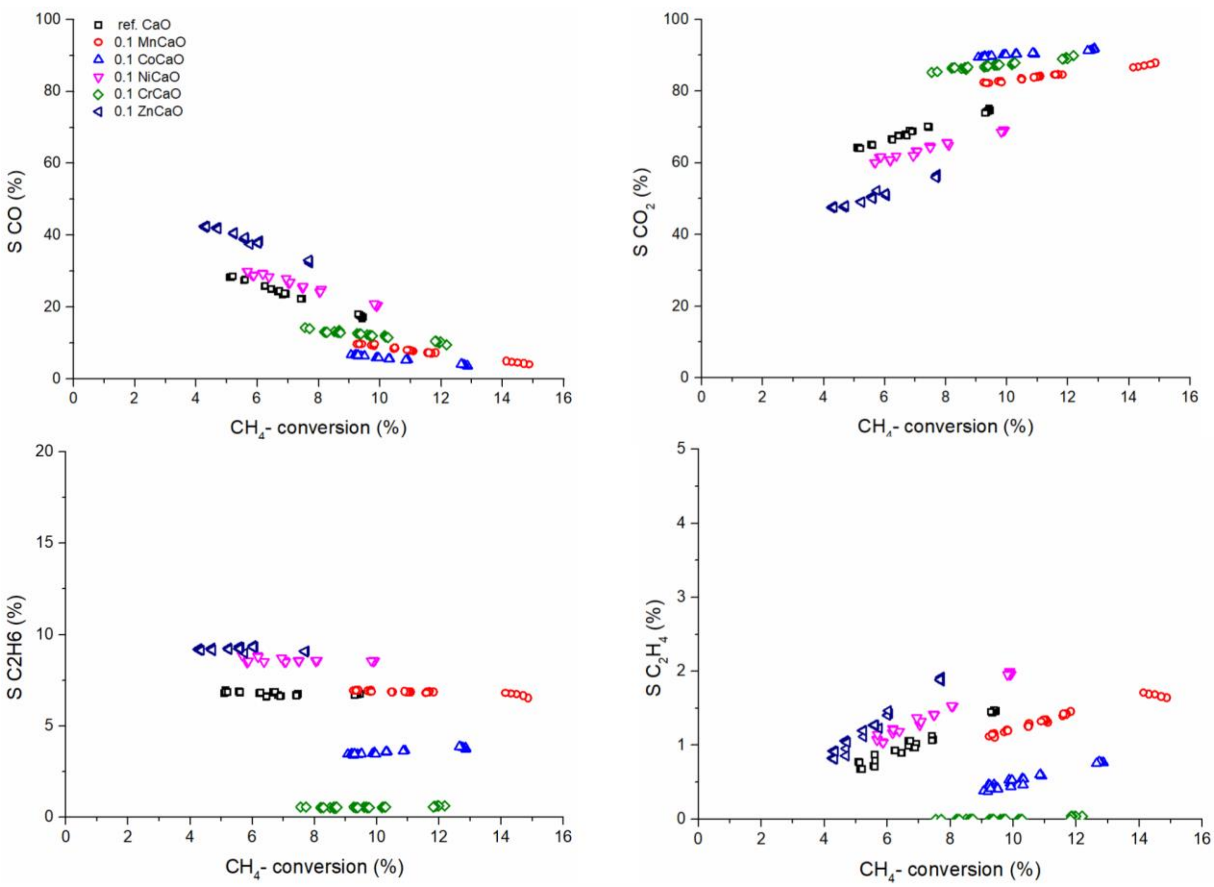

Figure S15: Dependence of OCM products by GHSV variation using different doped CaO catalysts. GHSV was varied by change of volume flow from 37.5 ml/min up to 200 ml/min.

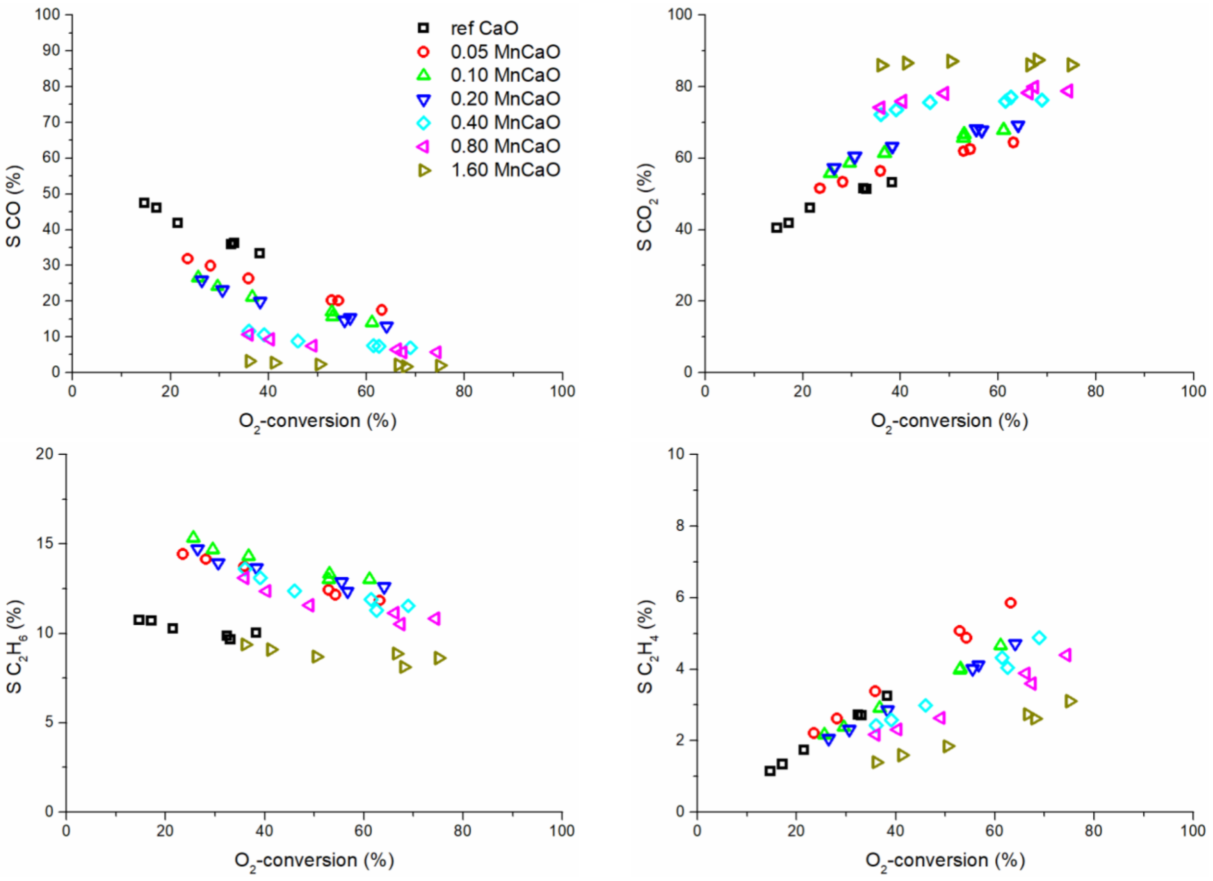

Figure S16: Dependence of OCM products by GHSV variation using Mn-doped CaO catalysts. GHSV was varied by change of volume flow from 37.5 ml/min up to 200 ml/min.

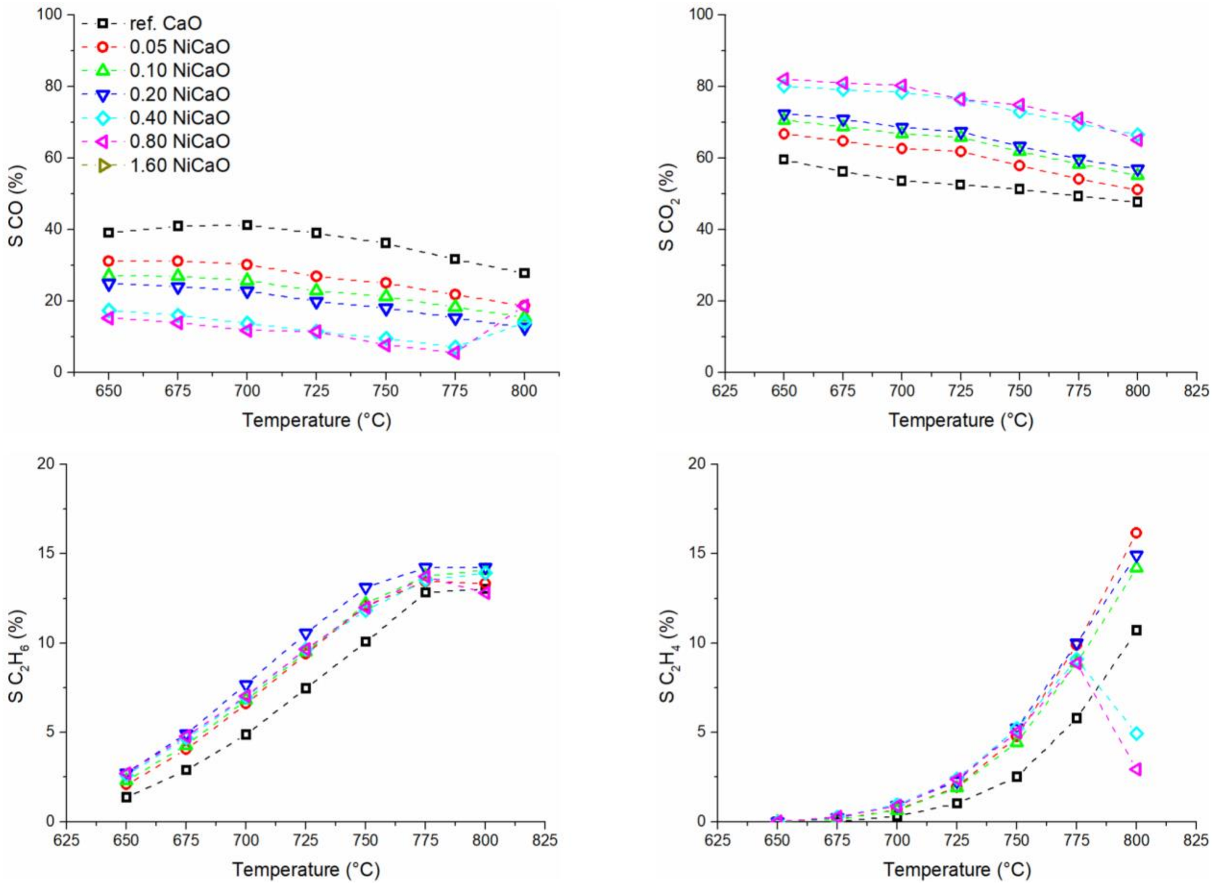

Figure S17: Temperature dependence of OCM products using Ni-doped CaO catalysts (50 mg carbonate precursor, 750 mg SiC, 50 ml/min 3:3:1 CH_4:N_2:O_2).

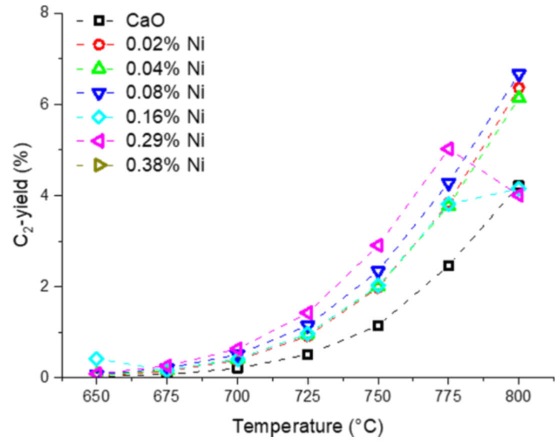

Figure S18: Temperature dependence of combined ethane and ethylene yield of nickel doped CaO catalysts (50 mg carbonate precursor, 750 mg SiC, 50 ml/min, CH_4:N_2:O_2 = 3:3:1).

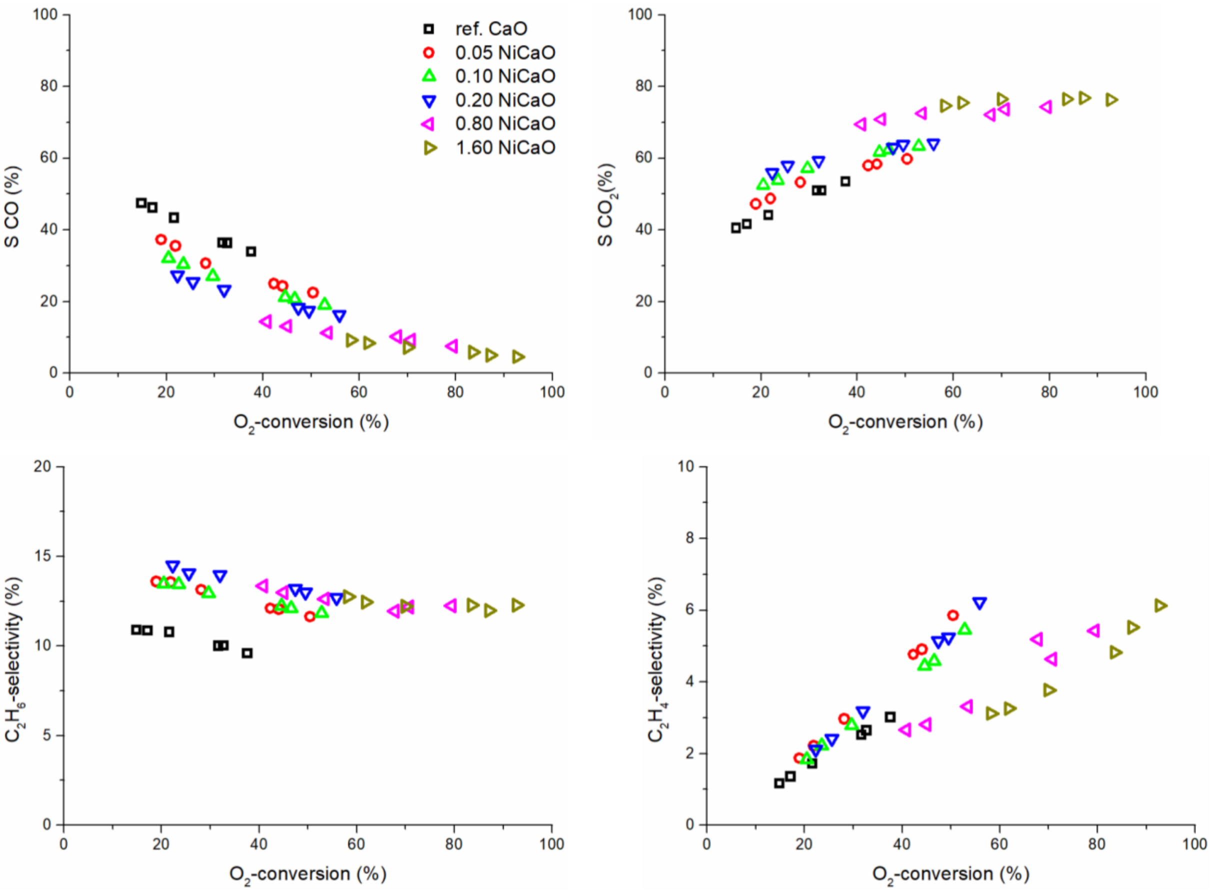

Figure S19: Dependence of OCM products by GHSV variation using Ni-doped CaO catalysts. GHSV was varied by change of volume flow from 37.5 ml/min up to 200 ml/min.

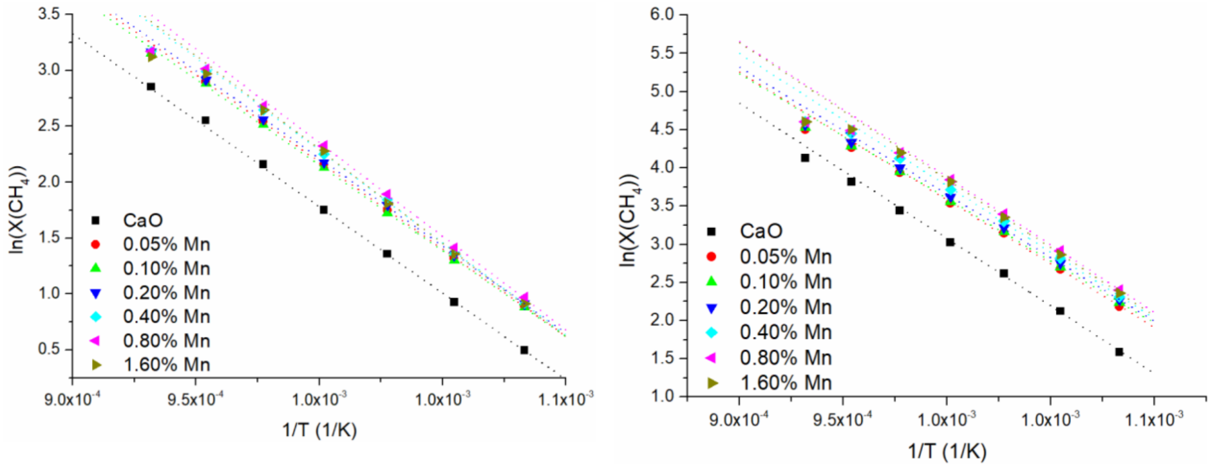

Figure S20: Arrhenius plots for Mn and Ni doping series.

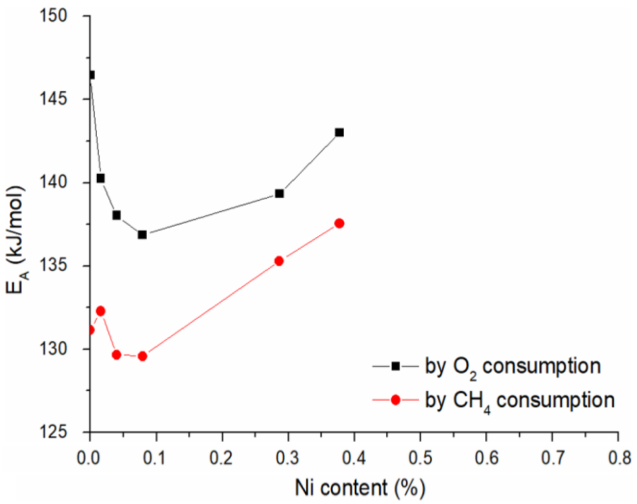

Figure S21: Calculated apparent activation energy of methane and oxygen consumption vs metal content of Ni doped CaO catalysts (50 mg carbonate precursor, 750 mg SiC, 50 ml/min 3:3:1 CH_4:N_2:O_2, the temperature range between 650 °C and 800 °C), lines are to guide the eye.

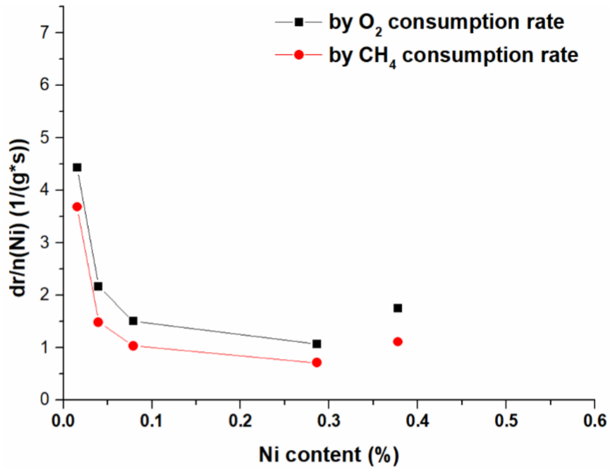

Figure S22: Change of oxygen and methane reaction rates normalized to amount of doping atoms vs total loading (50 mg carbonate precursor, 750 mg SiC, 50 ml/min 3:3:1 CH_4:N_2:O_2, 705 °C).
